# Nanocrystalline Ordered Mesoporous Co(OH)_2_ and Co_3_O_4_ Thin Films: Oxygen Evolution Reaction Activity from a Structural Properties Perspective

**DOI:** 10.1002/smsc.202500422

**Published:** 2025-12-13

**Authors:** Qingyang Wu, Stefan Lauterbach, Christian Dietz, Achim Alkemper, Lysander Q. Wagner, Helmut Schlaad, Jan P. Hofmann, Marcus Einert

**Affiliations:** ^1^ Surface Science Laboratory Department of Materials‐ and Geosciences Technical University of Darmstadt Peter‐Grünberg‐Strasse 4 64287 Darmstadt Germany; ^2^ Institute for Applied Geosciences, Geomaterial Science Technical University of Darmstadt Schnittspahnstrasse 9 64287 Darmstadt Germany; ^3^ Institute of Materials Science, Physics of Surfaces Technical University of Darmstadt Peter‐Grünberg‐Strasse 2 64287 Darmstadt Germany; ^4^ Institute for Physical Chemistry Justus‐Liebig University Giessen Heinrich‐Buff‐Ring 17 35392 Giessen Germany; ^5^ Center for Materials Research Justus‐Liebig University Giessen Heinrich‐Buff‐Ring 17 35392 Giessen Germany; ^6^ Institute of Chemistry University of Potsdam Karl‐Liebknecht‐Strasse 24‐25 14476 Potsdam Germany

**Keywords:** cobalt oxide, mesoporous, oxygen evolution reaction, thin films, water splitting

## Abstract

Design of nanostructured electrocatalysts is essential to improve the efficiency for driving the oxygen evolution reaction (OER) at low overpotentials. Mesoporous cobalt‐based thin films are prepared by dip‐coating and soft‐templating using the structure‐directing diblock copolymer poly(ethylene‐co‐butylene)‐block‐poly(ethylene oxide). Our temperature‐dependent study reveals how the calcination temperature affects the phase formation and development of the surface and bulk morphology of the catalysts. The crystallographic structure, surface composition, and development of the mesoporous framework were correlated with the OER activities. The increase in calcination temperature significantly impacts the nanoarchitecture, changing from an amorphous and dense structure, which is composed of Co(OH)_2_, to structurally intact and ordered mesoporous Co_3_O_4_ networks. The morphology of the mesoporous network (providing accessibility for the electrolyte), the overall surface area, and the presence of a nanocrystalline Co(OH)_2_ pre‐catalyst phase (allowing fast formation of electrocatalytically active species), collectively determine the OER activity. These structure–property relationships explain why Co(OH)_2_ films annealed at 250 °C show the lowest overpotential of 370 mV at 10 mA cm^−2^ and electrochemical stability in alkaline media. The development of the ordered mesoporous architectures in dependence on the annealing temperature demonstrates the importance of careful tailoring of the synthesis conditions to achieve optimized OER performance.

## Graphical abstract

It is demonstrated how the structural integrity of a mesoporous framework, providing accessibility for the electrolyte, the (electrochemical) active surface area, which predetermines the exposure of reaction sites, and the crystallinity, allowing fast surface reconstruction and formation of electrocatalytically active species, collectively impact the oxygen evolution reaction activity of Co(OH)_2_ and Co_3_O_4_ thin film electrocatalysts.

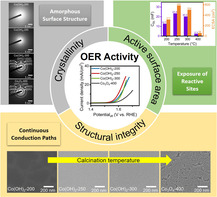

## 1 Introduction

Hydrogen has been demonstrated as a promising alternative energy vector and storage medium to replace conventional fossil fuels for sustainable and renewable energy supply. Water electrolysis is one valuable technique to produce highly pure and carbon‐neutral hydrogen. Generally, water splitting involves the hydrogen evolution reaction (HER) occurring at the cathode and the oxygen evolution reaction (OER) at the anode. HER in both acidic and alkaline media proceeds via a two‐electron transport process, while OER is considered as mechanistically more complex owing to the involvement of four electrons and protons, which need to be transferred. The lowest overpotentials of OER catalysis have been reported for noble metal oxides, such as iridium oxide (IrO_2_) and ruthenium oxide (RuO_2_) in acidic electrolytes,^[^ ^1^, ^2^ ^]^ and for NiFe‐based materials under alkaline reaction conditions.^[^ ^3^, ^4^, ^5^ ^]^ However, these aforesaid electrocatalysts were either costly in production and resource critical, or mainly unstable under OER conditions (e.g., iron segregation, peeling off from substrates).^[^ ^4^, ^6^ ^]^

As alternative, cobalt‐based electrocatalysts, such as spinel cobalt oxide (Co_3_O_4_) and cobalt hydroxide (Co(OH)_2_) are cost‐efficient, earth‐abundant, relatively stable in alkaline electrolytes and show competitive OER activity compared to other electrocatalytically active transition metal oxides (TMOs), for example, nickel oxide (NiO_*x*_), iron oxide (Fe_3_O_4_), or NiFeO_*x*_.^[^ ^7^, ^8^ ^]^ Co_3_O_4_ crystallizes in the cubic spinel structure (space group Fd$\bar{3}$m), which consists of Co^3+^ ions occupying the octahedral sites, coordinated by six oxygen atoms, and Co^2+^ ions are located at tetrahedral sites with four ligand oxygen atoms (considering a normal spinel configuration).^[^ ^9^, ^10^, ^11^ ^]^ Layered α‐ and β‐Co(OH)_2_ are polymorphs of Co(OH)_2_, where α‐Co(OH)_2_ has a larger interlayer spacing than β‐Co(OH)_2_, resulting in higher catalytic activity.^[^ ^12^ ^]^ Compared to other 3d transition metal hydroxides (e.g., Fe(OH)_2_ and Mn(OH)_2_), Co(OH)_2_ was found to have a better reactivity in alkaline OER owing to the optimum bond strength between the adsorbed OH^−^ and the active metal site.^[^ ^13^ ^]^ With the retention of morphology, Co(OH)_2_ can be thermally transformed into Co_3_O_4_.^[^ ^14^, ^15^ ^]^ Commercially available and particulate Co_3_O_4_, possessing a surface area of 52 m^2^ g^−1^, exhibits an overpotential of 451 mV at 10 mA cm^−2^ in 1 m KOH.^[^ ^16^, ^17^ ^]^ However, pristine Co_3_O_4_ suffers from poor intrinsic electronic conductivity, which vastly limits the OER activity.^[^ ^18^ ^]^ The limitation can be overcome, for example, by heteroatom doping,^[^ ^19^, ^20^, ^21^ ^]^ exposed facet manipulation,^[^ ^22^, ^23^, ^24^ ^]^ introducing oxygen vacancies,^[^ ^25^, ^26^ ^]^ and/or nanostructuring.^[^ ^17^ ^]^ Mesoporous TMO structures are of particular interest for catalytic applications, since mesoporous networks benefit from a larger internal contact area, allow for proper infiltration of the electrolyte, and therefore provide improved mass transport and shorter diffusion paths for effective charge transfer—parameters that collectively improve the OER performance.^[^ ^27^, ^28^, ^29^, ^30^, ^31^, ^32^ ^]^ It is worth noticing that there is a merit of mesostructuring of Co_3_O_4_ as thin films over the usage of nanoparticle‐based powders as electrocatalysts, since these mesoporous frameworks are anchored to a substrate, thus inhibiting the aggregation of particles, especially during operation, which would substantially reduce the number of exposed catalytically active sites.^[^ ^33^ ^]^ Research has been conducted on mesoporous Co(OH)_2_ for the application as capacitors or electrodes in Li‐ion batteries,^[^ ^15^, ^34^ ^]^ however only one report was found on mesoporous Co(OH)_2_ used as OER catalyst but in the form of powders.^[^ ^35^ ^]^

In this context, soft‐templating, based on classic sol–gel chemistry, provides a direct and versatile route to prepare mesostructured thin films.^[^ ^36^, ^37^, ^38^, ^39^ ^]^ Soft‐templating is connected to the evaporation‐induced self‐assembly concept,^[^ ^40^ ^]^ which allows for fabrication of mesoporous thin films via self‐assembly of the structure‐directing agent leading to formation of micelles and spontaneous assembly and condensation of inorganic reagents. Subsequently, the thermally‐induced removal of the organic template (structure‐directing agent) and the transformation of the amorphous into a nanocrystalline phase occurs upon calcination in air.^[^ ^27^ ^]^ However, the successful retention of mesoporous structures in practice is dependent on complex interactions in solution, for example, the mismatch of charges between precursors and surfactant.^[^ ^41^ ^]^ Several self‐assembly strategies have been reported, such as the surfactant‐salt assembly^[^ ^42^, ^43^ ^]^ or self‐assembly of stable liquid crystalline mesophases,^[^ ^41^, ^44^, ^45^ ^]^ to circumvent these drawbacks. For instance, Dag et. al. prepared mesoporous metal lithiate^[^ ^46^ ^]^ and metal titanate^[^ ^47^ ^]^ via molten‐salt‐assisted self‐assembly achieving pore sizes smaller than 10 nm.^[^ ^48^ ^]^ Yet, it requires two kinds of surfactants and high concentration of salts to form stable mesophases.^[^ ^49^ ^]^ Especially the use of KLE for the one‐step preparation of metal oxide thin film electrocatalysts by soft‐templating, possessing comparatively large mesopores (15−20 nm) and a structurally intact inorganic network, is therefore of interest for the OER in terms of charge carrier transport paths (electric conductivity) and improved accessibility of the electrolyte owing to larger mesopore sizes and an interconnected porous system.

Recently, a facile approach was reported by our group for the preparation of disordered mesoporous Co_3_O_4_ thin films by combining the commercially available triblock copolymer Pluronic F‐127^[^ ^50^ ^]^ and citric acid (CA), which forms a metal–CA complex that stabilizes the mesoporous structure during calcination.^[^ ^51^ ^]^ However, the study focused on the investigation of disordered mesoporous Co_3_O_4_ networks, which showed breakdown of the mesoporous network, and thus, the structural connectivity, resulting in the formation of discontinuous electronic conduction paths within the overall nanoarchitecture. The importance of structural integrity for migrating charge carriers within the pore walls was proven to be disadvantageous for migration of photoexcited electrons in mesoporous TiO_2_ photoanodes.^[^ ^52^ ^]^ Pluronic F‐127 was also reported to have a low segregation strength that cannot sustain the mesoporous framework upon calcination in order to reach a certain crystallinity.^[^ ^53^ ^]^ Poly(ethylene‐co‐butylene)‐block‐poly(ethylene oxide) (KLE) shows better templating properties than Pluronic F‐127 in terms of developing a mesoporous framework with interconnected crystalline pore wall domains.^[^ ^54^ ^]^ The use of KLE as structure‐directing agent during dip‐coating typically leads to the formation of periodically ordered mesopores, as it was demonstrated for various metal oxide compounds.^[^ ^53^, ^55^, ^56^, ^57^ ^]^ Larger mesopores can be produced with KLE as compared to Pluronic F‐127.^[^ ^58^ ^]^ Large‐mesopore catalysts have been reported to show higher OER activity due to better diffusion of the electrolyte and a facilitated ionic‐transport process.^[^ ^59^, ^60^ ^]^ Consequently, the influence of structural integrity on the electrocatalytic activity of mesoporous metal oxide thin films needs to be investigated to state, if these ordered mesoporous frameworks positively affect the charge carrier transfer at the interface. Furthermore, the impact of external experimental factors during/after dip‐coating, such as relative humidity, calcination temperature, and calcination time, still remains unrevealed and needs further investigation to understand how the evolving nanoscale framework influences the OER performance.

Hence, in this work, ordered mesoporous cobalt hydroxide and oxide thin films were fabricated by dip‐coating. By performing a temperature‐dependent study, the impact of structural and physicochemical properties of Co(OH)_2_ and Co_3_O_4_ on their electrochemical performance were systematically investigated and correlated with each other. An incremental transition of the crystallographic structure from cobalt hydroxide to cubic spinel oxide phase in dependence on the calcination temperature was observed. The data show that both a structurally intact mesoporous network, possessing large surface areas, and low annealing temperatures, favoring Co(OH)_2_ formation, predetermine the OER activity.

## 2 Results and Discussion

### 2.1 Structural Properties

**Figure 1:**
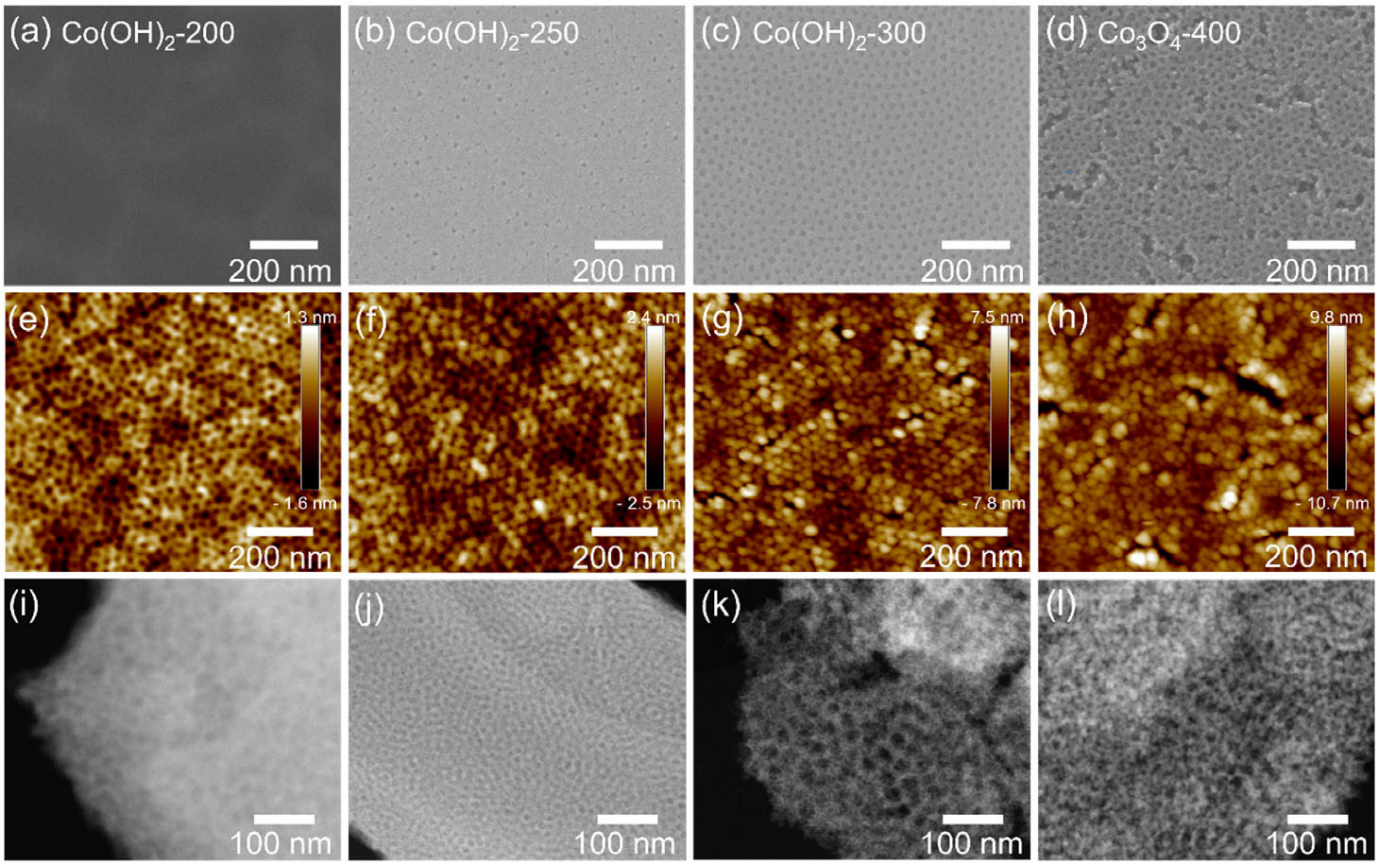
Temperature‐dependent investigations of the surface morphology of cobalt‐based thin films: a–d) SEM images; e–h) AFM images accomplished by amplitude modulation; i–l) dark‐field STEM images for each thin film calcined at either 200, 250, 300, or 400 °C for 10 min in air.

The surface structure and morphology are influential to the overall OER activity, as they affect the content and availability of active surface sites and the migration distances for charge carriers. Therefore, surface structures of cobalt‐based thin films were analyzed by scanning electron microscope (SEM). It is important to mention that the nomenclature for cobalt‐based thin films in the following is chosen based on the chemical surface composition obtained from X‐ray photoemission spectroscopy (XPS) and near‐edge X‐ray absorption fine structure (NEXAFS), since the surface structures typically control the OER performance.^[^ ^61^, ^62^ ^]^ The as‐prepared thin films were named as “Co(OH)_2_” or “Co_3_O_4_”, with a hyphen followed by the respective calcination temperature. The optimization of the solution recipe and the dip‐coating parameters (e.g., humidity, drying time, and withdrawal speed), allowed for the synthesis of reproducible samples in terms of bulk and surface morphologies. As shown in **Figure** **1**a–d, the SEM images illustrate the development of the mesoporous network in dependence of annealing temperatures. The surface morphology of Co(OH)_2_‐200 is mainly compact in nature, and no porous features were identified (Figure 1a). This can be related to the thermal decomposition behavior of the KLE template, which was examined to occur predominantly at 371 °C in N_2_, as shown in Figure S1a, Supporting Information and in literature.^[^ ^56^ ^]^ However in air, the onset decomposition temperature was measured to be ≈218 °C in Figure S1b, Supporting Information, which explains that the initial formation of ill‐defined nanopores start to appear at 250 °C (Figure 1b). For Co(OH)_2_‐250, the pores are randomly distributed at the surface. Some of the nanopores are not completely open, which seems reasonable because at this temperature the weight loss of polymer is ≈10% (Figure S1b, Supporting Information), meaning that the pores are still filled by the polymer. An ordered mesoporous architecture with pore diameters of 15–20 nm and crack‐free structures on the nano‐ and micrometer level were attained for the thin film calcined at 300 °C (Figure 1c). The intrinsic (e.g., concentration of precursors) and extrinsic parameters (such as the relative humidity and withdrawal speed) during dip‐coating, needed to be systematically optimized (see Experimental Section) to obtain ordered and crack‐free mesoporous structures. Additionally, it was found that the development of the mesoporous framework is also linked to the holding time during calcination at 300 °C (see Figure S2, Supporting Information). For example, the sample calcined for 5 min at 300 °C (Figure S2a, Supporting Information) displayed less microcracks and more pore ordering than the ones calcined for only 10 min (Figure 1c) and 30 min (Figure S2b, Supporting Information), respectively. This proves that these mesoporous films are metastable structures that are only stable within a certain temperature and time window. To investigate the impact of humidity on the formation of the mesoporous framework, the sample calcined at 300 °C for 30 min was prepared at 9.5% (Figure S2c, Supporting Information), 15% (Figure S2d, Supporting Information), and 25% relative humidity (Figure 1c). The samples prepared at 9.5% and 15% humidity showed a high density of (nano‐) cracks and poorly developed mesoporous networks, while at 25% humidity, an open mesopore architecture with the absence of any cracks was obtained. Based on this systematic analysis, in this study, a relative humidity of 25% and an annealing time of 10 min were chosen, as the thin films—processed under these conditions—possess the highest pore ordering and structural integrity. At an annealing temperature of 400 °C, thermally‐induced stress within the mesoporous Co_3_O_4_ network leads to formation of cracks (size varies from 30 to 100 nm) on the nanometer scale (Figure 1d). The pore wall domains and the average pore diameter increase with the rise of annealing temperature, which can be assigned to the complete decomposition of the polymer and the growth of nanocrystals induced by the calcination in air.^[^ ^38^, ^63^ ^]^ The average mesopore sizes of the samples, determined by evaluation of top‐view SEM images, are collected in **Table** **1**. The thicknesses of the thin films are determined to be 782 nm for Co(OH)_2_‐200, 764 nm for Co(OH)_2_‐250, 709 nm for Co(OH)_2_‐300, and 508 nm for Co_3_O_4_‐400 by profilometry (Figure S3, Supporting Information). The trend shows that with increasing calcination temperature the thickness decreases due to the decomposition of the polymer and densification of the inorganic framework.^[^ ^32^ ^]^

**Table 1:** The collection of mesopore size obtained by SEM, particle size by TEM, RMS roughness by AFM, crystallite size by XRD, *R*_ct_ determined by EIS, electrical conductivity (*σ*) determined by in‐line 4‐point‐probe measurements, Tafel slopes, *C*_DL_ values from the fitting results for EIS data, the current loss after CP performed in percentage, and overpotential at 10 mA cm^−2^ (η_10_).

Sample	Pore size by SEM [nm]	Particle size by TEM [nm]	Roughness by AFM [nm]	Crystallite size by XRD [nm]	* R*_ct_ [Ω]	*σ* [S cm^−1^]	Tafel slope [mV dec^−1^]	*C*_DL_ [mF]	Current loss by CP [%]	η_10_ [mV]
Co(OH)_2_‐200	–	–	0.40	–	9.4	797	72	12.7	1.1	390
Co(OH)_2_‐250	12–14	–	0.65	–	7.5	847	69	23.4	1.2	370
Co(OH)_2_‐300	15–20	6	1.81	–	12.2	1026	62	20.2	1.8	390
Co_3_O_4_‐400	>20	14	2.63	6	27.9	1495	100	2.3	3	440

As shown in Figure 1e–h, the topography of the samples’ surfaces was investigated by atomic force microscope (AFM) in the amplitude modulation mode at the scale of one micrometer. For Co(OH)_2_‐200 (Figure 1e), the ordered mesoporous surface morphology appears to be evident in the AFM micrographs, but not in the SEM and scanning transmission electron microscopy (STEM) images (Figure 1a or Figure 1i, respectively). It is important to note that the dark regions in AFM micrographs represent only a lower value in the apparent height, and do not necessarily indicate the presence of pores. Considering that cobalt carbonate initially forms at a minimum temperature of 160 °C and that KLE completely decomposes at ≈400 °C, the observed quasimesostructure may reflect a combination of residual carbonate and polymer templates. If this interpretation holds true, caution is needed when analyzing amplitude modulation topography images. This is because the apparent height may result from a combination of the true height and an additional “parasitic” height caused by differences in the mechanical properties between the “stiff” cobalt compound and the “soft” carbonate/polymer residues, which arise from varying tip indentation differences.^[^ ^64^, ^65^ ^]^ Because of the distinct usage of polymer and annealing time, the occurrence of nanocracks was postponed until 300 °C (Figure 1g). With major retention of the ordered porous structure, the nanocracks appear, which can be attributed to the volumetric shrinkage of the inorganic framework due to thermal stress.^[^ ^66^ ^]^ Up to 400 °C, the film exhibited more nanocracks with larger size, and the ordered structure seemed to be disturbed, which can be caused by the combined impact of conversion from carbonate to oxide, and the crystallization of the oxide phase. The root‐mean‐square (RMS) roughness of thin films was determined to be 0.40 nm for Co(OH)_2_‐200, 0.65 nm for Co(OH)_2_‐250, 1.81 nm for Co(OH)_2_‐300, and 2.63 nm for Co_3_O_4_‐400, with AFM 3D images shown in SEI Figure S4, Supporting Information. These values are aggregated in Table 1. The increasing RMS roughness confirms the topography changes with the rise of calcination temperature, and such an increase in roughness may also related to the transition from Co(OH)_2_ to Co_3_O_4_, which has also been reported by Liu et al.^[^ ^14^ ^]^

The cobalt‐based thin films were further characterized by STEM in dark‐field mode and transmission electron microscopy (TEM) in bright‐field mode, as shown in Figure 1i–l and Figure S5, Supporting Information, respectively. It can be seen that at a relatively low temperature such as 200 °C (Figure 1i), a “prepore” structure has formed, and been embedded in an amorphous matrix, where no nanocrystallites are present. Up to 250 °C, this “prepore” feature still dominates, with an ordered mesoporous network identified from Figure 1j and Figure S5b, Supporting Information, indicating the start of pore formation, agreeing with the results of SEM and AFM. For Co(OH)_2_‐300, the mesopores become more prominent, and agglomeration of particles is observed, appearing as brighter spots in STEM (Figure 1k) and darker areas in TEM (Figure S5c, Supporting Information), respectively. The pore development between 200 and 300 °C corresponds well to the AFM and SEM images. For Co_3_O_4_‐400, the STEM images exhibit a distorted mesoporous network, accompanied with the growth and agglomeration of the nanoparticles (shown in Figure 1l and Figure S5d, Supporting Information). Overall, the temperature‐dependent structural characterization by SEM, AFM, and TEM demonstrate that the calcination temperature has a major impact on the formation of the mesoporous network and crystalline structure.

**Figure 2:**
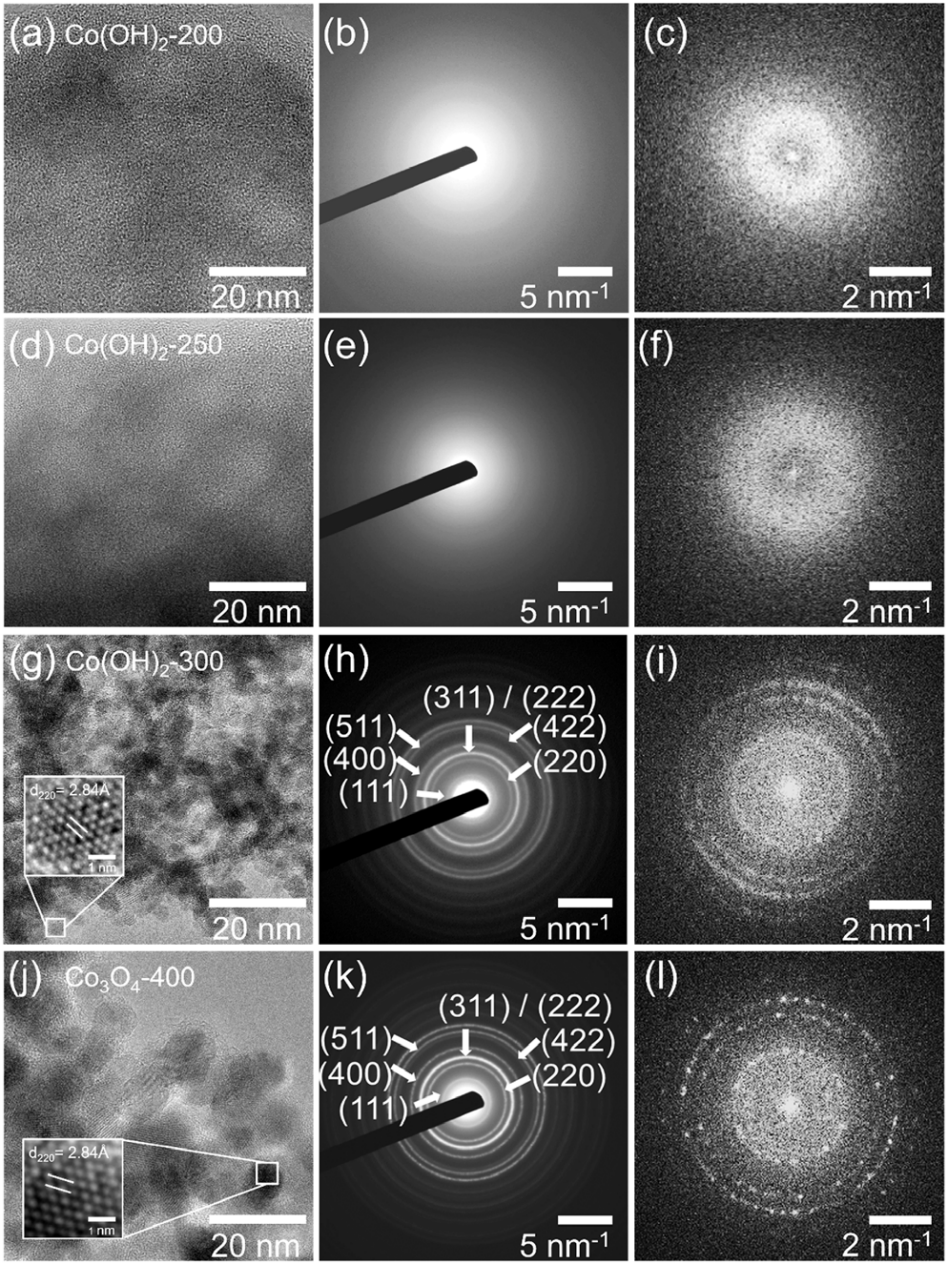
a,d,g,j) HR‐TEM images of cobalt‐based thin films; b,e,h,k) SAED patterns from larger bulk areas indexed (h,k) in agreement with the cubic spinel phase, and c,f,i,l) patterns generated by FFT from the whole image area. Inserted iFFT images in (g) and (j) reveal atomic distances for the (220) lattice planes in the spinel phase.

High resolution‐TEM (HR‐TEM) and selected area electron diffraction (SAED) analysis were carried out (**Figure** **2**) to evaluate the nanostructure and crystallinity of cobalt‐based thin films. As indicated in Figure 2a,d, Co(OH)_2_‐200 and Co(OH)_2_‐250 do not show pronounced diffraction patterns/rings in the SAED (Figure 2b,e) images, however a weak and diffuse ring is observed in the fast Fourier transform (FFT) picture (Figure 2c,f). This indicates that the bulk structures of Co(OH)_2_‐200 and Co(OH)_2_‐250 are mainly composed of amorphous domains, which is in accordance with Figure 1, however might already contain nanocrystalline Co(OH)_2_ features at the surface (see discussion later). We note that the absence of any observed nanocrystallinity in Co(OH)_2_‐200 and Co(OH)_2_‐250 (no diffraction rings in Figure 2b,e) might also—at least partially—be attributed to coexistence of the KLE polymer, which is stable up to 300 °C according to thermogravimetry analysis (TGA) (see Figure S1, Supporting Information). In contrast, Co(OH)_2_‐300 shows discrete sharp rings in the SAED pattern, which can be indexed to the (111), (220), (311)/(222),(400), and (511) lattice planes, as shown in Figure 2h. These observations are in line with the bright‐field TEM image (Figure S5c, Supporting Information) and dark‐field STEM image (Figure 1k) demonstrating both nanocrystalline domains with mesoporous structures and partially amorphous features with prepore morphologies. An inverse FFT (iFFT) filtered image from the boxed area shows a interplanar spacing of 2.84 Å (Figure 2g), which corresponds well to the cubic spinel Co_3_O_4_ crystal system with lattice spacing d_220_ = 2.8 Å in agreement with literature.^[^ ^24^ ^]^ Although, the interplanar spacing of Co(OH)_2_ can also vary from 2.4 to 4.4 Å, when the samples are present as different polymorphs,^[^ ^67^, ^68^, ^69^ ^]^ the interplanar spacing of 2.01 Å, which matches with the (400) lattice plane in the cubic spinel phase and is missed (for the d‐spacing) of Co(OH)_2_, implies that Co_3_O_4_ was partially formed in the bulk of sample Co(OH)_2_‐300. The HR‐TEM images of Co_3_O_4_‐400 (Figure 2j), depicts the presence of nano‐/mesopores embedded in a nanocrystalline Co_3_O_4_ matrix, and this observation is supported by the STEM (Figure 1l) and SEM/AFM data (Figure 1d,h), collectively proving the formation of an ordered mesoporous framework. The iFFT image of the selected area in Figure 2j also demonstrates a d‐spacing of 2.84 Å, indicative for the formation of the cubic spinel structure of Co_3_O_4_‐400 suggesting complete phase transition. The particle sizes of Co(OH)_2_‐300 and Co_3_O_4_‐400 in TEM were determined to be on average 6.1 and 14.1 nm, respectively (collected in Table 1).

Assessing the specific surface area of the mesoporous cobalt‐based thin film was accomplished by krypton physisorption at 77 K, as shown in Figure S6, Supporting Information. Following the Brunauer–Emmett–Teller (BET) model and the procedure in the Experimental Section for estimating the sample mass, a surface area of 30 m^2^ g^−1^ is obtained for the Co_3_O_4_‐400 sample, indicating the presence of both a mesoporous surface and bulk morphology. Although mesoporous oxide thin films of comparable pore structure can typically possess higher specific surface areas,^[^ ^57^ ^]^ the lower value is reasonable in this case taking the high density of the crystalline oxide (6.4 cm^3^ g^−1^) into account, which reduces the specific surface area (i.e., normalized to the sample mass).^[^ ^70^ ^]^

**Figure 3:**
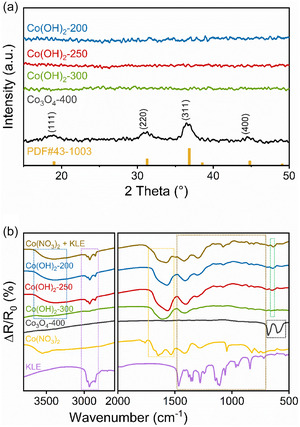
a) XRD pattern measured in grazing‐incidence mode with reference bars presenting Co_3_O_4_ JCPDS no. 43‐1003, and b) IRRAS spectra of cobalt oxide and hydroxide thin films calcined at various temperatures.

To further characterize the crystallographic bulk structure and to confirm the TEM data, grazing‐incident X‐ray diffraction (GIXRD) analysis was conducted (**Figure** **3**a). No diffraction peaks were identified for Co(OH)_2_‐200 and Co(OH)_2_‐250, indicating a lack of bulk crystallinity in accordance with the TEM data. It is worth mentioning that Co(OH)_2_‐300 shows no diffraction peaks in the patterns (olive line in Figure 3), which were however observed by SAED in Figure 2h. This can be explained by the difference in detection size between X‐ray diffraction (XRD) and SAED, as XRD gives more information from the complete bulk of the material, while SAED focuses on very small areas and is more sensitive to nanocrystals due to the higher interaction of electron with matter. It could also be attributed to the size of crystallites in Co(OH)_2_‐300 that is too small for XRD, which can be related to the well‐developed ring pattern without detectable discrete spots in SAED (Figure 2h). For Co_3_O_4_‐400, four diffraction peaks were identified at 19.0, 31.1, 36.6, and 44.4°, which can be assigned to (111)‐, (220)‐, (311)‐, (400)‐planes, respectively. The data are consistent with the reference of a cubic Co_3_O_4_ spinel phase (JCPDS no. 42‐1003). XRD patterns show no additional peaks of any Co, CoO, or CoOOH side phases. The crystallite size of the Co_3_O_4_‐400 sample was determined by applying the Scherer equation to the (311) peak, and was calculated to be ≈6 nm.

To exclude the presence of secondary phases, the cobalt‐based thin films were examined with Raman spectroscopy, as shown in Figure S7a, Supporting Information. The spectra of Co(OH)_2_‐200 and Co(OH)_2_‐250 show no obvious Raman peaks, which could be attributed to the thin layer of Co(OH)_2_ and the amorphous nature of Co(OH)_2_‐200 and Co(OH)_2_‐250, as SAED (Figure 2b,e) and XRD (Figure 3a) suggest. Compared to the Raman spectra of Co(OH)_2_ from literature^[^ ^71^, ^72^, ^73^ ^]^ that report to have multiple distinct peaks between 400 and 600 cm^−1^, the spectrum of Co(OH)_2_‐300 shows a broad feature between 450 and 650 cm^−1^ (Figure S7a, Supporting Information). The broad feature could result from the long‐range disorder owing to the amorphous structure of Co(OH)_2_‐300 (as proved by XRD in Figure 3a), and also reported for amorphous Ta_2_O_5_.^[^ ^74^ ^]^ The pronounced peak at 692 cm^−1^ (gray area at right in Figure S7a, Supporting Information) is found in the spectrum of amorphous Co(OH)_2_,^[^ ^71^ ^]^ while it also appears as a shoulder peak in the spectrum of Co_3_O_4_‐400.^[^ ^73^ ^]^ It indicates that the phase transition from Co(OH)_2_ to Co_3_O_4_ occurs between 300 and 400 °C, as confirmed by TEM (Figure 2 and Figure S5, Supporting Information). For Co_3_O_4_‐400, five Raman peaks are observed at 471,193, 516, 611, and 677 cm^−1^, corresponding to the E_g_, F_2g_, and A_1g_ bands of the cubic Co_3_O_4_ spinel phase, respectively.^[^ ^73^, ^75^, ^76^ ^]^ The A_1g_ peak at 677 cm^‐1^ is attributed to the vibrational mode mainly determined by the oxygen ions at octahedral sites, while the two weak peaks of E_g_ (471 cm^−1^) and F_2g_ (516 cm^−1^) are formed by the combined vibration of tetrahedral and octahedral oxygen atoms.^[^ ^77^, ^78^, ^79^ ^]^ The Raman spectra of cobalt‐based thin films without substrate subtraction are shown in Figure S7b, Supporting Information in SEI. We note that the determined crystallographic structures by TEM and XRD are solely related to the bulk structure of the thin films and the surface composition is analyzed by XPS in the following section.

To support the structural data derived by XRD and Raman spectroscopy and to determine the functional surface groups, infrared reflection absorption spectroscopy (IRRAS) measurements were conducted for films on IR‐reflective fluorine‐doped tin oxide (FTO) substrates, and the spectra are shown in Figure 3b. To demonstrate the temperature‐dependent changes in bond vibrations associated with the used organic surfactant (KLE) and metal precursors (e.g., nitrate precursor), thin films were prepared by dip‐coating with the solutions (under identical conditions as described in Experimental Section) containing cobalt nitrate (Co(NO_3_)_2_) alone, KLE alone, and the mixture of both (Co(NO_3_)_2_ + KLE), respectively, and then calcined at 100 °C for 10 min to evaporate the solvents. For Co(OH)_2_ samples, the weak band at around 644 cm^−1^ (green frame) is assigned to Co–OH vibrations.^[^ ^80^, ^81^ ^]^ There is a broad absorption band appearing from 3300 to 4000 cm^−1^ (blue frame) in the spectra of Co(OH)_2_ corresponding to the O—H stretching from lattice hydroxide.^[^ ^82^, ^83^ ^]^ The band found in the range of 1600–1650 cm^‐1^ originates from the H—O—H bending of adsorbed H_2_O.^[^ ^84^ ^]^ The bands between 2800 and 3000 cm^−1^ (violet frame), which can be found for the ‐CH_2_ stretching mode in PEO copolymers,^[^ ^85^ ^]^ appear in Co(OH)_2_‐200, Co(OH)_2_‐250, Co(NO_3_)_2_ + KLE and KLE, while they are extremely low in intensity in the spectrum of Co(OH)_2_‐300 and absent in the one of Co_3_O_4_‐400. This indicates that major decomposition of KLE has occurred after 300 °C, corresponding well with the TGA data (Figure S1, Supporting Information). It is reported that the broad band between 1508 and 1733 cm^−1^ (yellow frame) can be related to both residual NO_3_^−^,^[^ ^81^ ^]^ stable up to 250 °C,^[^ ^83^ ^]^ and/or the carbonate formed at low temperatures.^[^ ^66^ ^]^ However, the absence of both a pronounced band between 1450 and 1480 cm^−1^ and a weaker one between 700 and 800 cm^−1^ reported for cobalt carbonate in literature,^[^ ^86^ ^]^ is an indication for the presence of nitrates as dominant species. The vibrational bands between 700 and 1481 cm^−1^ (brown frame) can be ascribed to O—H bending and C—H bending stemming from the KLE template.^[^ ^87^ ^]^ The specific assignments for the vibrational bands in the IR‐spectrum of KLE are listed in Table S1, Supporting Information. The decreasing intensity of all peaks in this region for increasing calcination temperatures suggest the decomposition of nitrate‐ and polymer‐related species, which is a reasonable finding for this temperature range. For Co_3_O_4_‐400, two primary longitudinal optical vibrational modes of the Co_3_O_4_ spinel phase appear for wavenumbers between 500 and 1000 cm^−1^ (Figure 3b; black frame).^[^ ^88^ ^]^ The band at 682 cm^−1^ corresponds to the Co(II)—O stretching vibration on tetrahedral sites, while the one at 600 cm^−1^ is associated with Co(III)—O stretching vibration on the octahedral sites.^[^ ^81^, ^89^ ^]^ The absence of any other absorption bands in the spectrum of Co_3_O_4_‐400 allows the assumption of both the complete transition from Co(OH)_2_ to Co_3_O_4_ phase and the degradation of KLE upon calcination, observations which are in good accordance with the XRD (Figure 3a) and TGA (Figure S1, Supporting Information) results. Note that band locations and band intensities in IRRAS can differ from those in transmittance spectra, depending on parameters, such as the thickness and/or the optical constants of the films.^[^ ^90^ ^]^

### 2.2 Surface Analysis

**Figure 4:**
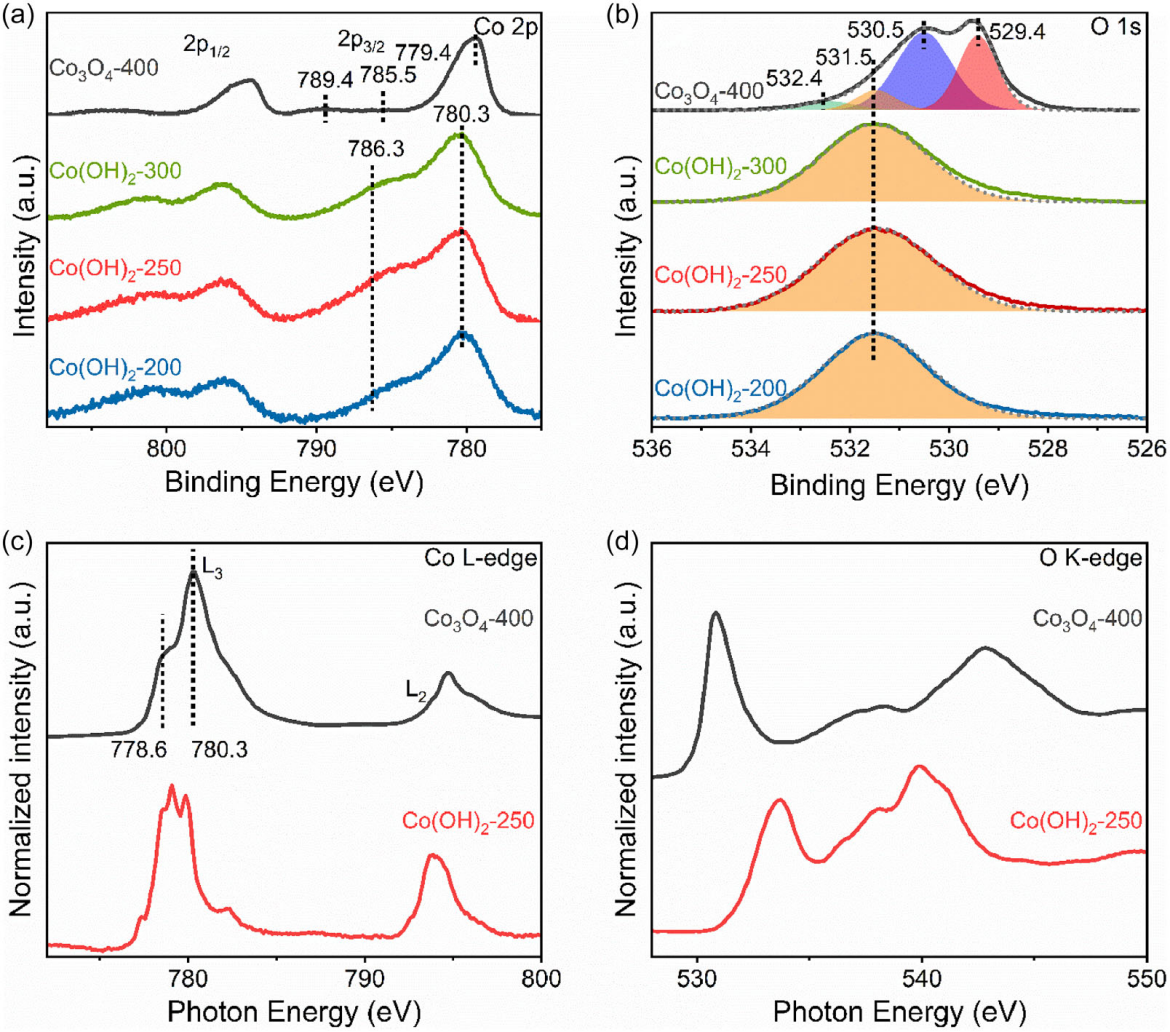
XPS spectra of a) Co 2p, b) O 1s photoemission lines, and NEXAFS spectra of c) Co L‐edge and d) O K‐edge of cobalt oxide/hydroxide thin films.

The chemical oxidation states and binding configurations of the elements on the surface of Co_3_O_4_ and Co(OH)_2_ thin films were analyzed through XPS. All spectra were referenced to the aliphatic carbon C1s signal at binding energy (BE) of 284.8 eV, and the shift of Co(OH)_2_ spectra due to charging were manually offset based on the position of aliphatic carbon in Co_3_O_4_‐400 and literature values.^[^ ^91^ ^]^ The survey and C 1s spectra are shown in Figure S8, S9, Supporting Information, respectively. Since identical chemical information is provided by both spin‐orbit splitting components Co 2p_1/2_ and Co 2p_3/2_,^[^ ^92^ ^]^ the analysis to **Figure** **4**a focuses on Co 2p_3/2_ core level spectra because of the higher intensity. The primary peak, located between 779.0 and 780.5 eV, is observed in both Co_3_O_4_ and Co(OH)_2_ thin films.^[^ ^93^ ^]^ Compared to the spectra of Co(OH)_2_ thin films obtained at 200, 250, and 300 °C, the peak for the Co_3_O_4_–400 sample is asymmetric in shape, which is characteristic for the spinel oxide with mixed oxidation state of Co^2+^ and Co^3+^. It is found to be shifted to a lower BE of 779.4 eV. Such shift in BE was also found in literature.^[^ ^25^, ^73^ ^]^ Corresponding to the primary peaks, in the higher BE region between 785.0 and 786.5 eV, satellite photoemission lines can be found. The satellite peaks arise due to multielectron interaction processes such as multiplet splitting, charge transfer, or shake‐up. In Figure 4a, the Co(OH)_2_ thin films can be distinguished from Co_3_O_4_ by the pronounced broad multielectron excitation (i.e., “shake‐up”) satellite peaks at 786.3 eV, which are reported to be found in Co(OH)_2_, but not for Co(OOH), and additionally, the intensity of this peak is decreased in the spectrum of spinel Co_3_O_4_ accompanied with a shift to lower BE.^[^ ^94^ ^]^ In Co_3_O_4_, the satellite peaks indicate that both valence states exists, and the weak satellite feature at 789.4 eV is closely linked to the existence of Co^3+^,^[^ ^93^ ^]^ while the one at 785.5 eV is assigned to Co^2+^ ions, which were more frequently observed in rock‐salt type CoO instead of Co_3_O_4_.^[^ ^95^ ^]^ The O 1s spectra including the data fitting, reveal information about the various components in the compound, as shown in Figure 4b. The O 1s spectra of Co(OH)_2_ thin films have a prominent peak centered at 531.5 eV, which could be assigned as a combination of contribution from bound hydroxide groups (OH^−^) and carbon‐oxygen species (see the violet peak in Figure S9, Supporting Information).^[^ ^72^, ^73^, ^96^, ^97^ ^]^ In contrast to the O 1s spectrum of Co_3_O_4_ reported in literature,^[^ ^98^ ^]^ which typically shows the most pronounced single peak at around 530.0 eV, for Co_3_O_4_‐400, the O 1s spectrum (Figure 4b; black data) depicts two intensive peaks at 529.4 and 530.5 eV that can be attributed to lattice O^2−^ and hydroxide groups, respectively. According to the Pourbaix diagram of cobalt,^[^ ^99^, ^100^, ^101^ ^]^ the feature appearing at higher BE (530.5 eV) most likely have contribution from undercoordinated oxygen formed in ambient atmosphere that later transforms into hydroxide as it is energetically more favorable.^[^ ^98^, ^102^ ^]^ It is less possible that this doublet peak is indicative for the formation of CoOOH,^[^ ^73^, ^98^ ^]^ although in Co_3_O_4_ both Co^2+^ and Co^3+^ can be either oxidic or hydroxidic, and additionally Co^3+^ could also form oxyhydroxide species.^[^ ^73^, ^97^, ^98^ ^]^ The other two peaks at 531.5 and 532.4 eV can be assigned to either hydroxyl groups and low‐coordinated defective oxide groups (including oxygen vacancies), and adsorbed water or C—O species (Figure S9, Supporting Information) on the surface, respectively.^[^ ^25^, ^103^ ^]^ It cannot be excluded that amorphous CoO_*x*_ has formed beneath the surface in all Co(OH)_2_ thin films, because XPS only investigates the oxidation state of the atoms on the surface layers of a material (few nanometers), while Raman spectroscopy, XRD, and TEM provide information about the bulk structure due to a higher penetration depth (in the μm resp. nm range).^[^ ^97^ ^]^ In general, the XPS spectra confirm that all samples can be related to the formation of Co(OH)_2_ and spinel‐type Co_3_O_4_ surface phases.^[^ ^104^, ^105^ ^]^

NEXAFS measurements were performed for the representative hydroxide Co(OH)_2_‐250 and oxide Co_3_O_4_‐400 samples (Figure 4c,d) to verify the observation of XPS and to receive a more detailed picture about the composition. As the detection depth of NEXAFS (about 3 to 4 nm) is slightly larger than for conventional XPS, the overall information can also consist of contributions from pore walls in the bulk of the materials. The Co L‐edge spectra of all thin films in Figure 4c consist of two main regions, namely the L3‐ and L2‐edge, respectively. Both edges originate from the transition from 2p to 3d orbitals of a transition metal, and are split into two by spin‐orbital coupling.^[^ ^106^ ^]^ The L3‐edge provides information about the valence state of the metal atom and the hybridization of metal‐ligand orbitals. The spectrum of the Co(OH)_2_‐250 thin film presents a multiplet feature that could originate from Jahn–Teller distortion^[^ ^107^ ^]^ Co(OH)_2_ is subjected to and which causes the presence of multiple electronic states. The splitting of peaks can also be attributed to the distortion of the crystal structure in Co(OH)_2_‐250.^[^ ^108^ ^]^ For the spinel Co_3_O_4_‐400 thin film, the spectrum shows doublet peaks in L3, indicating the presence of Co^2+^ located on tetrahedral sites and Co^3+^ on octahedral sites, at 778.6 and 780.3 eV, respectively. The energy shift of 1.7 eV corresponds well to the literature value,^[^ ^109^ ^]^ which was also found for other 3d TMOs.^[^ ^110^ ^]^ The change in morphology (e.g., nanostructuring) of the sample might induce a variation in peak intensity in NEXAFS.^[^ ^108^ ^]^ Perspective work will focus on elucidation of these aspects. The O K‐edge NEXAFS was carried out to identify the unoccupied states. As displayed in Figure 4d, in the spectrum of Co_3_O_4_‐400, the single peak at 530.7 eV strongly indicates a low‐spin Co^3+^ (t_2g_^6^) ground state as Co^3+^ is more covalent in nature, that is, with a stronger oxygen feature, compared to Co^2+^.^[^ ^111^, ^112^ ^]^ The trend of the shift in *hv* of the main peak in the O K‐edge spectra (from 533.2 eV to 530.8 eV) for calcination temperature of 250 °C (Figure 4, red line) to 400 °C (Figure 4, black line) is in contrast to that of the major peaks (at 779.0 eV) in the Co L3‐edge spectrum. This indicates increased valence states with increasing calcination temperature, which is a reasonable finding. In summary, both XPS and NEXAFS reveal the chemical surface states of the electrocatalyst thin films, which experience a transition from Co(OH)_2_ to Co_3_O_4_ upon increasing calcination temperature.

### 2.3 OER Activity

**Figure 5:**
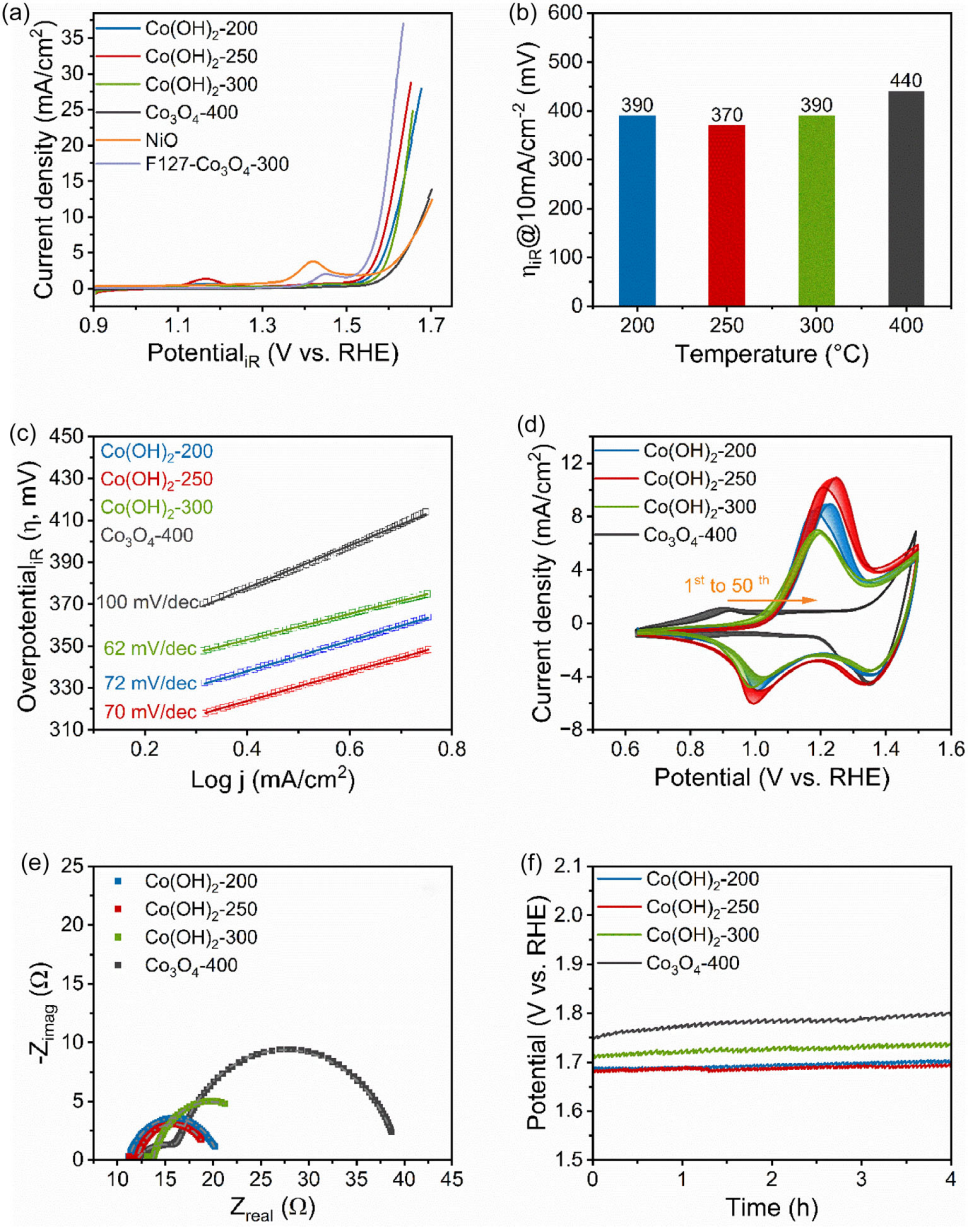
Electrochemical characterization of mesoporous Co(OH)_2_ and Co_3_O_4_ thin films. a) LSV curves collected at a scan rate of 10 mV s^−1^, b) column plot of overpotentials obtained by LSV at 10 mA cm^−2^ as function of the calcination temperature, c) Tafel plot with the corresponding fitting, d) CV curves measured with scan rate of 100 mV s^−1^ for 50 cycles, e) Nyquist plots determined by EIS at 1.6 V, and f) CP testing recorded at 10 mA cm^−2^ for 4 h in 1 m KOH.

To evaluate the OER performance of the sol–gel‐derived cobalt‐based thin films, as‐prepared Co(OH)_2_ and Co_3_O_4_ thin films were analyzed and evaluated by geometric linear sweep voltammetry (LSV) in 1 m KOH in the potential range between 0.9 to 1.7 V versus RHE (**Figure** **5**a). In Figure 5a, the onset potentials were found to be at 1.53 V for Co(OH)_2_‐200, 1.51 V for Co(OH)_2_‐250, 1.54 V for Co(OH)_2_‐300, and 1.55 V versus RHE for Co_3_O_4_‐400, indicating the highest OER activity for Co(OH)_2_‐250 and lowest for Co_3_O_4_‐400. This observation is in line with the determined overpotentials derived from LSV curves at 10 mA cm^−2^, which decline from 390 (Co(OH)_2_‐200) to 370 mV (Co(OH)_2_‐250), and then increase to 390 mV (Co(OH)_2_‐300). The highest value was identified with 440 mV for Co_3_O_4_‐400 (Figure 5b). A nickel oxide (NiO) thin film from Gallenberger et al.^[^ ^113^ ^]^ was added as reference compound (orange curve in Figure 5a) for comparison to Co_3_O_4_‐400. Since the surface structure and electrochemical active surface area (ECSA) has major impact on the OER activity, comparison of the geometric OER activity of catalysts is intricate. For proper comparison, mesoporous NiO thin films with comparable ECSA and also prepared by soft‐templating show an overpotential of more than 500 mV even after the activation of 5 h potentiostatic treatment in 1 m KOH at a potential of 1.75 V versus RHE.^[^ ^114^ ^]^ We note that NiO can be activated into hydroxides or oxyhydroxides by preconditioning in OER the region.^[^ ^115^, ^116^ ^]^

In the work by Kaiser et al.^[^ ^116^ ^]^ electrochemically nonconditioned Ni(OH)_2_ showed overpotentials of 345 mV versus RHE at 10 mA cm^−2^ in 1 m KOH, which is a comparable OER activity as was found for the underlying Co(OH)_2_ samples. Structurally‐related mesoporous Co_3_O_4_ thin films, also synthesized through soft‐templating however with Pluronic F‐127 and calcined at 300 °C for 30 min (Figure 5a and Figure S10, Supporting Information),^[^ ^50^ ^]^ demonstrate an overpotential of 340 mV in 1 m KOH. This OER activity is lower than the one of KLE‐templated Co_3_O_4_‐400, indicating that both the surface morphology and crystallinity, including the size of nanocrystallites, has major impact on the OER activity. We note that mesoporous Co(OH)_2_ thin films were prepared—to the best of our knowledge—for the first time via dip‐coating and soft‐templating, exhibiting good OER activity. Common overpotentials of Co(OH)_2_ are predominantly reported between 380 and 390 mV at 10 mA cm^−2^.^[^ ^117^ ^]^ It is known that the surface of Co_3_O_4_ and Co(OH)_2_ can (partially) transform into the CoOOH phase in the non‐OER region (in 1 m KOH), and CoOOH is capable of further transforming into CoO_2_.^[^ ^14^, ^118^ ^]^

To understand the reaction mechanisms upon OER on mesoporous Co(OH)_2_ and Co_3_O_4_ thin films, the OER kinetics were interpreted by the Tafel slope (Figure 5c). It provides the potential, which is required, to increase the current by a factor of 10, and thus, presented in the unit of mV dec^−1^. A low Tafel slope value is an indication of an active catalyst, as a smaller overpotential is required to reach a higher current density.^[^ ^119^ ^]^ One of the most used mechanism was proposed by Krasil'shchikov for common metal oxides (including Co_3_O_4_) in alkaline OER^[^ ^120^ ^]^

(1) $$M^{*}+{\text{OH}}^{-}\to M-\text{OH}+e^{-},\;b=120\text{ mV}\;{\text{dec}}^{–1}$$

(2) $$M-\text{OH}+{\text{OH}}^{-}\to M-O^{-}+H_{2}O,\;b=60\text{ mV}\;{\text{dec}}^{–1}$$

(3) $$M-O^{-}\to M-O+e^{-},\;b=45\text{ mV}\;{\text{dec}}^{–1}$$

(4) $$\text{2M}-O^{-}\to 2M^{*}+O_{2},\;b=19\text{ mV}\;{\text{dec}}^{–1}$$

For the underlying samples, Tafel slopes were determined to be 72 mV dec^−1^ for Co(OH)_2_‐200, 70 mV dec^−1^ for Co(OH)_2_‐250, 62 mV dec^−1^ for Co(OH)_2_‐300, and 100 mV dec^−1^ for Co_3_O_4_‐400. The specific values of the Tafel slopes for each sample were collected in Table 1. For Co_3_O_4_‐400, the highest Tafel slope (100 mV dec^−1^) among all samples indicates the least facilitated OER kinetics. The intermediate value between 120 and 60 mV dec^−1^ suggests that the surface adsorbed species (M—OH) predetermines the oxygen evolution kinetics in the reaction.^[^ ^121^, ^122^, ^123^ ^]^ Shinagawa et al. proposed a similar mechanism—as was found for the underlying Co(OH)_2_ thin films—that the adsorption of hydroxide anions in alkaline environment predetermines the formation of metal oxyhydroxide (MOOH) as the second step upon the OER.^[^ ^124^ ^]^ Indeed, the Tafel slopes of the Co(OH)_2_ thin films collectively show values between 60 and 70 mV dec^−1^ independent of the calcination temperature. A Tafel slope close to 60 mV dec^−1^ indicates that the rate‐determining step in OER is an activation controlled one‐electron transfer, which is valid to all Co(OH)_2_ films according to Shinagawa's theory.^[^ ^125^, ^126^ ^]^ Among all samples, Co(OH)_2_‐300 possess the lowest slope of 62 mV dec^−1^, which in principle indicates the most facilitated kinetics during the OER process. However, Co(OH)_2_‐300 exhibits a higher charge transfer resistance (*R*_ct_) and overpotential (390 mV) in comparison to the best‐performing Co(OH)_2_‐250 (370 mV, see Table 1). This difference is most likely related to the fact that the derivation of the Tafel slope is located in the “kinetic control” regime (current density of 3–6 mA cm^−2^),^[^ ^127^ ^]^ in which the well‐developed mesoporous architecture observed for Co(OH)_2_‐300 (see Figure 1), facilitates the OER kinetics by better infiltration of the electrolyte leading to more accessible surface sites for OH^−^ adsorption.^[^ ^128^ ^]^ In contrast, at 10 mA cm^−2^, where *R*_ct_ was measured, the system enters the “mix” regime and the Tafel slope is impacted by multiple nonkinetic factors, such as an increased generation of gas.^[^ ^129^ ^]^ According to the Butler–Volmer equation, exchange current densities (j_0_) of the hydroxide thin films follows the sequence: Co(OH)_2_‐250 > Co(OH)_2_‐200 > Co(OH)_2_‐300, which are directly linked to the *R*_ct_ according to Ohm's law. Hence, *R*_ct_ is the reflection of the combined effect of both the exchange current density and Tafel slope at this specific overpotential.

Cyclic voltammograms (CVs) were measured in 1 m KOH at a scan rate of 100 mV s^−1^ for 50 cycles to identify the occurring redox reactions. As indicated in Figure 5d, pronounced redox peaks were observed in the potential range between 0.6 and 1.5 V versus RHE. The most noticeable anodic peaks for Co(OH)_2_‐200, Co(OH)_2_‐250, and Co(OH)_2_‐300 are observed in the range of 1.1 to 1.3 V versus RHE (also visible in LSV curves in Figure 5a), which can be assigned to the oxidation of Co^2+^ to Co^3+^ (Co(OH)_2_ + OH^−^ → CoOOH + H_2_O + e^−^).^[^ ^12^, ^117^, ^130^ ^]^ The corresponding cathodic (back) reaction occurs at ≈1.0 V versus RHE, and the declined intensity of both the anodic and cathodic peak over the course of 50 cycles indicates that less oxidation of Co(OH)_2_ species to CoOOH occurs.^[^ ^13^, ^130^, ^131^ ^]^ The trend of the variation in intensity of the Co^2+^/Co^3+^ peak for the Co(OH)_2_ thin films, representing the amount of active Co^2+^ species, is in accordance with LSV data. A variation in the positions of oxidation peaks among all samples was found, which can be likely assigned to the distinct surface morphologies and the varying degree of crystallization (e.g., grain sizes) in dependence of the annealing temperature that lead to the changing amount of active surface sites available for the OER.

To characterize the resistance of the charge transfer at the electrolyte/catalyst‐interface, electrochemical impedance spectroscopy (EIS) was applied at 1.6 V versus RHE within a frequency range of 100 kHz to 0.1 Hz. Figure 5e presents the EIS data in the form of Nyquist plots fitted based on a Randles circuit model, which consists of a parallel connection between *R*_ct_ and a constant phase element, together in a series link to the uncompensated resistance in the system (*R*_s_). The attained data are listed in Table 1. All samples possess comparable *R*_s_ values (12 ± 1 Ω), which is reasonable since all samples were measured in the same cell setup and in 1 m KOH. Interestingly, *R*_ct_ shows the same trend as the overpotentials derived from LSV data (Figure 5a and Table 1): *R*_ct_ decreases from 9.4 Ω for Co(OH)_2_‐200 to 7.5 Ω for Co(OH)_2_‐250, and then increases again to 12.2 Ω for Co(OH)_2_‐300. The generally low *R*_ct_ values prove the facilitated transfer of charge carriers from the electrocatalyst to electrolyte. The Nyquist plot of Co_3_O_4_‐400 shows two semicircles, which is commonly seen for porous electrode systems since the capacitance of the system partially arises from the contribution of the conductive substrate, which is exposed to the electrolyte due to the porous nature of the metal oxide electrode.^[^ ^132^ ^]^ Compared to our previous work on Co_3_O_4_ thin films prepared with Pluronic F127 (*R*_ct_ = 3.5 Ω), the *R*_ct_ of Co_3_O_4_‐400 is higher (27.9 Ω), which probably results from the higher crystallinity and the presence of cracks at the nanoscale (see Figure 1d), interrupting the electric conduction paths. Interestingly, two pronounced semicircles were observed for the Co_3_O_4_‐400 sample, which can be assigned to the larger crystallite sizes.^[^ ^27^ ^]^ The grain size‐dependent electrical conductivity of mesoporous ceria‐zirconia solid solutions was characterized via EIS by Hartmann et al.^[^ ^133^ ^]^ revealing the presence of only one semicircle for crystallite sizes between 4 and 12 nm (same size range as for the underlying Co_3_O_4_‐400 sample). The electrical conductivities (*σ*) of cobalt‐based thin films were determined to be 797 S cm^−1^ for Co(OH)_2_‐200, 847 S cm^−1^ for Co(OH)_2_‐250, 1026 S cm^−1^ for Co(OH)_2_‐300, and 1495 S cm^−1^ for Co_3_O_4_‐400, respectively, gathered in Table 1. The increasing electrical conductivity can be assigned to the development of mesoporous structure offering an increased interface density that facilitates better electron transport.^[^ ^27^, ^133^, ^134^ ^]^

The ECSA is another important parameter that quantifies the surface area, which is catalytically active in an electrochemical process. The ECSA can be determined from the ratio of the double layer capacitance (*C*_DL_) and the specific capacitance (*C*_s_) of the studied electrode material. Here for *C*_s_, a generally utilized value in alkaline electrolytes of 40 μF cm^−2^ is applied, taken from the report of McCrory et al.^[^ ^135^ ^]^ The *C*_DL_ values were accomplished by fitting of the EIS data based on the Randles circuit model (Figure 5e). The *C*_DL_ and derived ECSA values are plotted against the calcination temperatures of cobalt‐based thin films, as shown in Figure S11, Supporting Information. Co(OH)_2_‐250 exhibited the highest *C*_DL_ (23.4 mF), followed by Co(OH)_2_‐300 (20.2 mF), Co(OH)_2_‐200 (12.7 mF), and Co_3_O_4_‐400 (2.3 mF), which corresponds to the ECSA of 585 cm^2^ for Co(OH)_2_‐250, 504 cm^2^ for Co(OH)_2_‐300, 317 cm^2^ for Co(OH)_2_‐200, and 58 cm^2^ for Co_3_O_4_‐400. The data agree well with the temperature‐dependent morphological changes observed by electron microscopy (Figure 1). The second lowest surface area was found for Co(OH)_2_‐200 since the pores are still blocked by nondegraded KLE and the surface is not accessible, while at 250 °C a porous network starts to form with comparatively small nanopores in the range of 12 to 14 nm. This explains the Co(OH)_2_‐250 sample shows the highest ECSA. At 300 °C annealing temperature, the mesopores start to grow (see Figure 1 and 2), and thus the ECSA slightly decreases. After calcination at 400 °C, a fully open mesoporous architecture has formed with complete decomposition of the polymer template. As a consequence, the lowest ECSA appears for Co_3_O_4_‐400 (58 cm^2^), which is reasonable, since the sample possess the largest mesopores (among all control samples), which are above 20 nm, and shows a large amount of cracks (see Figure 1d), additionally reducing the density of mesopores, and thus, the overall surface area.

As the electrochemical stability of an electrocatalyst should be tested for practical applications, chronopotentiometry (CP) was conducted at 10 mA cm^−2^ in 1 m KOH for 4 h. As shown in Figure 5f, stable performances were observed for all thin films. Accordingly, the catalytic activity loss within this time frame was determined to be 3% for Co_3_O_4_‐400, 1.8% for Co(OH)_2_‐300, 1.2% for Co(OH)_2_‐250, and 1.1% for Co(OH)_2_‐200 (Co_3_O_4_‐400 > Co(OH)_2_‐300 > Co(OH)_2_‐250 > Co(OH)_2_‐200). This trend shows that the electrochemical stability of cobalt hydroxide and oxide films is an inverse function of the calcination temperature most likely owing to facilitated surface reconstruction for amorphous samples resulting in a stable surface structure. Although literature has reported that a crystalline Co_3_O_4_ thin film undergoes reversible amorphization in the OER region, and then recrystallizes when cycling back to the relaxing potential,^[^ ^136^ ^]^ the mesoporous structure of Co_3_O_4_‐400 sustains after performing CP analysis (Figure S12, Supporting Information). It is reported that the high degree of pore ordering, in principle, allows for better compensation of induced stress and strain to the porous network.^[^ ^50^, ^137^ ^]^ In conclusion, it was found that especially the crystal phase at the surface, rather than the mesopore arrangement and geometry, predetermine the electrochemical stability of mesoporous cobalt hydroxide and oxide thin film electrocatalysts.

**Figure 6:**
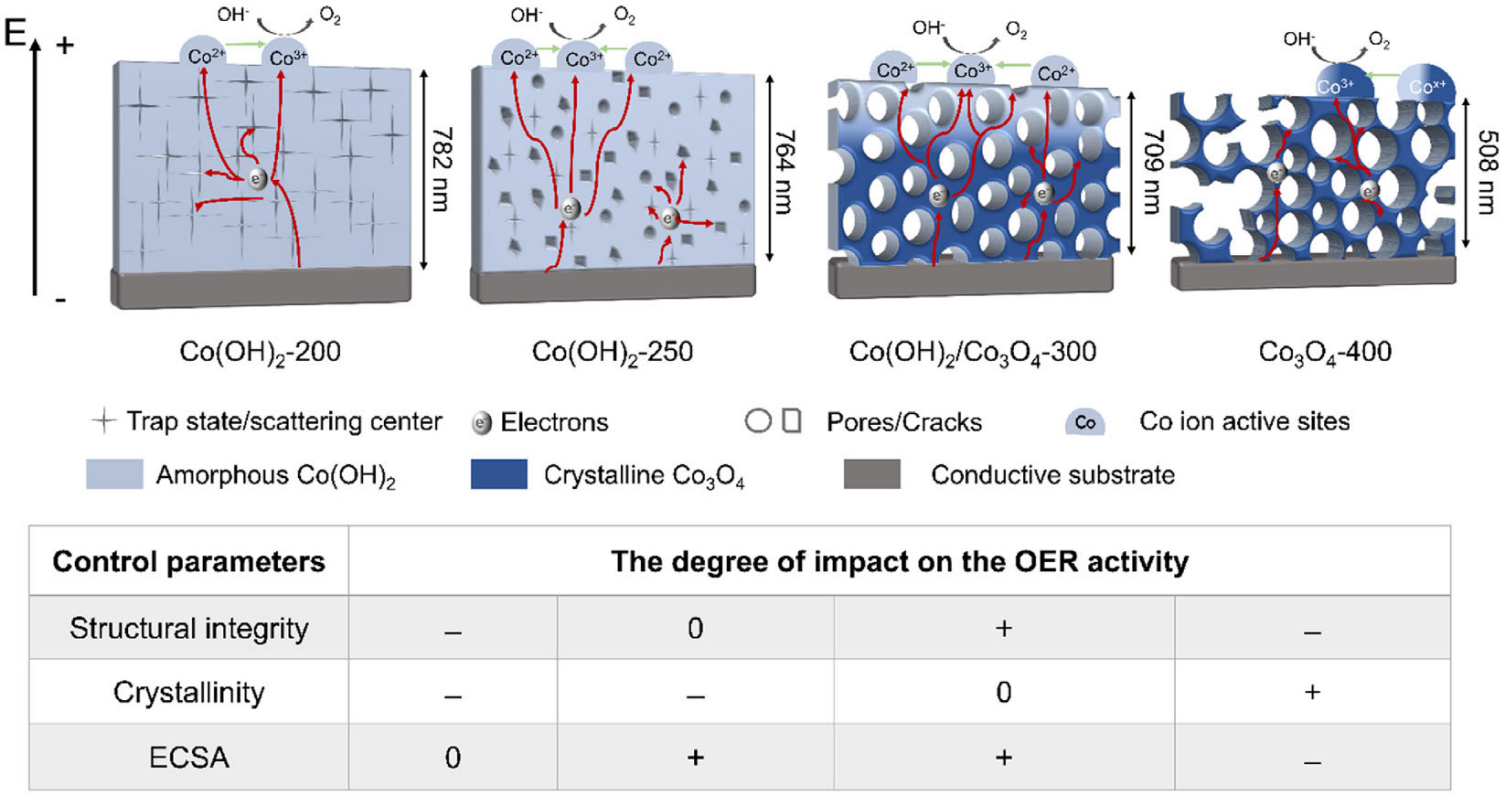
Schematic drawing of the charge carrier conduction paths throughout the Co(OH)_2_‐200, Co(OH)_2_‐250, Co(OH)_2_‐300, and Co_3_O_4_‐400 thin film in presence of an electric field. The control parameters structural integrity, crystallinity, and ECSA are attributed to the sketch and to which degree they impact the overall OER performance with + being strong/high, 0 being neutral, and – being weak/low.

To discuss and interpret the OER performance of the samples, three structural control parameters have to be considered, which are the structural integrity on the nanoscale (ensuring continuous and interconnected transport paths, and thus good electronic conductivity), the crystallinity of the sample (determining the kinetics of the surface reconstruction, and thus the formation of electrocatalytically active species), and the ECSA (presenting the total amount of reaction sites, and thus the surface reactivity). Note that Co(OH)_2_‐300 is labeled as Co(OH)_2_/Co_3_O_4_‐300 in **Figure** **6**, indicating that the bulk is composed of Co_3_O_4_ and the surface of Co(OH)_2_ (in accordance with Figure 2). With respect to the best‐performing Co(OH)_2_‐250 sample, the OER activity can be mainly assigned to 1) the only slightly developed mesoporous network, providing however, the highest (internal) surface area among all control samples due to the presence of small nanopores, and, at the same, 2) the preservation of the structurally intact nanoarchitecture with interconnected pore wall domains forming a continuous transport route for migrating charge carriers (see Figure 6, and 3) the amorphous nature of the surface allowing for facile transformation into catalytically active surface species (also illustrated by the highest oxidation peak in Figure 5d representing the largest amount of available Co^2+^ ions). For amorphous Co(OH)_2_‐200, no mesopores are present/visible (see Figure 1a and 6) and the stabilized carbonaceous (polymer) phase, as a result of the annealed KLE, might act as recombination sites for charge carriers decreasing the electronic conductivity (see Table 1), and hence the OER performance. Interestingly, Co(OH)_2_‐200 and Co(OH)_2_‐300 demonstrate the same overpotentials. Two major effects are responsible for that: by increasing the annealing temperature, on the one hand, the mesoporous structure advances (Figure 1 and 6) and the polymer degradation proceeds (positive for OER), on the other hand, however, the crystallinity increases at the same time, as observed by SAED (Figure 2), resulting in an energetically more stabilized surface structure (from a crystallographic perspective), which is less adaptive and prone to self‐reconstruction and possess less active sites (negative for OER).^[^ ^138^ ^]^ It has been reported that such in‐situ self‐reconstruction promotes the formation of electroactive amorphous species.^[^ ^139^ ^]^ A thermally‐induced creation of nano‐ and microsized cracks (see Figure 1 and 6) and pronounced crystallinity (see Figure 2 and 3a) are observed for Co_3_O_4_‐400 samples. These cracks and voids interrupt electronic conduction paths and cause a reduction of the ECSA. Additionally, proper infiltration of the electrolyte into the mesoporous network is hindered, and thus, efficient mass transport, resulting in decreased OER activity (Figure 5b).^[^ ^17^ ^]^ This study shows that the calcination temperature of sol–gel‐derived cobalt‐based oxides have to be chosen and tailored carefully, adjusting the interplay of several control parameters, impacting the OER performance opposingly. The nature of the surface structure, the overall surface area, and preservation of the mesoporous framework of cobalt hydroxide thin films has been found to predetermine the overall OER activity (Figure 6). However, and importantly, the accessible surface area and type ofelectrocatalytically active species seem to have the most dominant impact on the OER performance of mesoporous metal oxide thin films, since disordered mesoporous Co_3_O_4_ films with disconnected pore walls, and thus, low degree of structural integrity of the inorganic network showed lower overpotentials compared to the highly ordered mesoporous and structurally intact materials investigated in this work.^[^ ^50^ ^]^

## 3 Conclusion

In this work the influence of the calcination temperature on the structural properties and OER performance of soft‐templated cobalt hydroxide and cobalt oxide thin films, prepared by dip‐coating, was investigated. The temperature‐dependent characterization toward the surface morphology and topography, crystallography, and elemental composition were carried out for the cobalt‐based electrocatalysts by combining numerous physicochemical characterization techniques. The surface morphology was found to change substantially when increasing the calcination temperature from 200 to 400 °C in air. At 200 °C, the organic template was still present in the thin film, possessing an amorphous, nonporous, and dense structure, while between 250 and 300 °C the mesopores start to develop owing to decomposition of the structure‐directing agent. The structurally most intact mesoporous cobalt hydroxide network was formed through calcination at 300 °C for 10 min. The bulk structure of all samples calcined up to 300 °C were all determined to be amorphous as confirmed by XRD, Raman spectroscopy and TEM, while surface analysis by XPS and NEXAFS demonstrated that the surface phase was composed of cobalt hydroxide. Electron diffraction analysis showed that the cubic spinel phase (Co_3_O_4_) started to form in the bulk at 300 °C, however still containing a Co(OH)_2_ surface as confirmed by XPS. At 400 °C, the sample crystallizes as Co_3_O_4_ spinel structure in the bulk and at the surface, which were composed of nanoparticles (≈6 nm in diameter) intermingled with ordered and spherically shaped mesopores. When investigating the samples as electrocatalyst for the OER, Co(OH)_2_ annealed at 250 °C demonstrated the lowest overpotential of 370 mV at 10 mA cm^−2^ in 1 m KOH. The electrocatalytic stability tests at 10 mA cm^−2^ presented stable oxygen evolution for at least 4 h for all samples. The OER performance of mesoporous cobalt hydroxide and oxide thin films were elucidated to be dependent on three main control parameters, which are 1) the crystallographic structure controlling the transformation into the active oxyhydroxide species and determining the concentration of electrocatalytically active species at the surface (Co^3+^ versus Co^2+^), 2) the structural integrity represented by retention of the mesoporous framework upon annealing, providing interconnected conduction paths, and thus, good electronic conductivity, and 3) the electrochemically accessible surface area, predetermining the number of electrocatalytically active sites. This work shows the importance of optimizing the preparation conditions of sol–gel‐based cobalt oxide and hydroxide thin film electrocatalyst for enhancement of their OER performance, which might be further improved by creation of structurally intact inorganic networks containing smaller mesopores (<20 nm) for increase of the surface area.

## 4 Experimental Section

4.1

4.1.1

##### Reagents and Materials

All chemicals except from the polymer were of analytical grade and were used without further purification. Cobalt nitrate hexahydrate (Co(NO_3_)_2_·6H_2_O, 99.999%), and ethanol (EtOH, ≥99.8%) were purchased from Sigma‐Aldrich. CA (≥ 99.5%) and potassium hydroxide (KOH, 1 mol L^−1^) were purchased from Carl Roth. The polymer template poly(ethylene*‐*co‐butylene)‐block‐poly(ethylene oxide) (Kraton liquid‐block‐PEO, KLE) with an average molar mass of 7.8 kg mol^−1^ and 41 wt% PEO was synthesized as described elsewhere.^[^ ^54^ ^]^

##### Synthesis of KLE‐Templated Co(OH)_*2*_ *and Co*_*3*_ *O*_*4*_

The formula of the solution for dip‐coating was adapted from Eckhardt et al.^[^ ^51^ ^]^ In principle, 276 mg Co(NO_3_)_2_·6H_2_O, 91.3 mg CA were dissolved in 1 mL EtOH, while 45 mg KLE were dissolved in a mixture of 0.2 mL EtOH and 1.1 mL of double‐filtrated Millipore water. The mass fraction of KLE diblock copolymer is set to be ≈30 wt% with respect to the formed spinel oxide.^[^ ^140^ ^]^ Both solutions had been stirred using a magnetic stirrer for at least 30 min until a homogeneous solution was obtained. Both were then mixed dropwise, and subsequently stirred for 1 h to form a clear, transparent, and uniform solution. Prior to dip‐coating, all substrates, including silicon (100) wafers (n‐type, resistivity <3 mΩcm) and FTO glasses, were initially cleaned with EtOH and acetone, and thereafter all of the FTO substrates were treated by an UV Ozone cleaner (L2002A3, Ossila) for 10 min to remove possible organic contamination on the surface.

Dip‐coating on substrates was conducted with a dip‐coater (L2006A1‐EU, Ossila), with the moving arm enveloped by a self‐made plastic chamber, in which the relative humidity was controlled at 25% during the dip‐coating process in order to have a homogenous deposition and low volume‐shrinkage of the thin films. The dip‐coating was applied with a constant withdrawal speed between 2 and 8 mm s^−1^. After coating, the samples were dried in the chamber with controlled humidity for 4 min, and immediately transferred into a muffle furnace (Nabertherm LT3/11) with preheating at 125 °C. The preheating process lasted for at least 15 min for each sample, and then the furnace was heated to 200 °C with a ramp of 10 °C min^−1^ and the temperature was held for 1 h. The temperature was then raised to the given values (250 °C/300 °C/400 °C) with the rate of 10 °C min^−1^, and finally maintained at target temperature for 10 min.

##### Characterizations

Investigations of the surface morphologies of cobalt compound thin films were carried out via SEM on a Philips XL30 FEG, with acceleration voltages of 30 kV and working distance of 3–4 cm. The pore size was determined using the software Scandium. The thickness was determined using a Bruker profilometer (Dektak XT) with a scan rate of 30 μm s^−1^ and a stylus force of 3 mg. The topography of the samples was analyzed using an AFM (Brucker, Santa Barbara, CA, type: Dimension Icon) in the amplitude modulation mode with PPP‐FMAuD cantilever (NanoAndMore GmbH, Wetzlar, Germany). In this mode, the tip‐sample distance is varied such that the oscillation amplitude of the cantilever remains constant while the sample surface is tracked. We set the working amplitude to ≈60% of the free amplitude A_0_, (A_0_ ≈ 200 nm). The cantilever's spring constant (1.6 N m^−1^) was determined using the thermal noise method,^[^ ^141^ ^]^ and a resonance frequency of 71.966 kHz was measured for the cantilever, which has a guaranteed tip radius of less than 10 nm as specified by the manufacturer. The scan speed of the tip was set to 2 μm s^−1^. We calibrated the inverse optical lever sensitivity of the cantilever using a stiff sapphire sample. By pressing the tip against the surface, the amplitude reduction detected by the photodiode (in the unit of volts) was correlated with the relative motion (in the unit of nanometers) of the z‐piezo, yielding a sensitivity factor of *S* = 137 nm V^−1^ in our case. The roughness was calculated using Bruker Nanoscope analysis software. To correct for sample tilt and mitigate thermal drift which is typically unavoidable during imaging under ambient conditions, topography images were first‐order flattened. For the measurements in TEM, a pretreatment of samples is required. Flakes of the thin films were scraped off from the silicon substrates using a razor blade, and collected in a vial. After adding 2 mL of EtOH, they were dispersed in an ultrasonic bath for 20 s. The dispersion was set aside for another 20–30 s to allow the larger particles to settle down. Two to three droplets of the upper part of the dispersion solution were dropped on a carbon‐coated copper grid (holey type, Plano GmbH). After evaporating EtOH, the samples were coated with a thin carbon film (carbon coater MED 010, Bal‐Tec AG) to eliminate charging from the incident electron beam in the TEM. Examination of the samples were performed in the TEM and STEM mode in a FEG TEM (JEM2100F, JEOL Ltd). The particle size was determined using the average value of the measured size of 30 particles from TEM images. Krypton physisorption isotherms of the cobalt‐based thin films were recorded at 77 K with an Autosorb iQ by Quantachrome Instruments (Boynton Beach, FL, USA). The data were evaluated with the ASiQwin software with a multipoint evaluation method based on the BET model. Transferring the surface area into a specific value requires the sample mass, which was estimated from the density and volume of the oxide/hydroxide. The latter was calculated from the coated area and the film thickness, taking a typical porosity (reducing the overall sample volume) of 50% into account. All samples were degassed at 200 °C for 6 h prior to measurement to remove attached gases and water. GIXRD patterns of samples under Cu Kα radiation (*λ* = 1.5405 Å) were recorded by a Rigaku Smartlab diffractometer in the 2*θ* range of 10°–50° using a step size of 0.01°. The XRD pattern were smoothed using Savitzky–Golay method with 50 points of window width, and the baseline was subtracted with 1st and 2nd derivative. Raman spectra were acquired with a Senterra Raman microscope from Bruker with a 532 nm laser with 100x objective lens. The power of the incident laser was set to be 5 mW with a slit of 50 × 1000 μm^2^, a 3–5 cm^−1^ resolution (1200 grating). The scans were implemented for 30 s integration time and 20× coadditions, and spectra were recorded in the range of 150–800 cm^−1^. The background of all spectra was subtracted by the spectrum of a clean, uncoated FTO substrate recorded with the same measurement parameters, and the intensity was normalized according to the minimum and maximum of each individual spectrum. IRRAS was performed using a Bruker Vertex 80v (SNo 1309) spectrometer equipped with a wide LN‐MCT D315/B (InfraredAssociates Inc. ID316‐M) detector. The samples were investigated under the pressure of 1.66 hPa with extra 5 min pump time and placed in reflection geometry under grazing incidence of 80°. The tests were carried out in the range of 4000 to 500 cm^−1^. The background of spectra was subtracted using a clean, uncoated FTO substrate. XPS and NEXAFS measurements were conducted under the beamline 1.1‐P: PM3^[^ ^142^ ^]^ at BESSY II synchrotron in Berlin, which offers the photo energy ranging from 150 to 1800 eV. The Solid−Liquid‐Interface Analysis System (SOLIAS) endstation has been used and spectra were measured at normal emissions using a Phoibos (SPECS) energy analyzer with 1D delay line detector.^[^ ^143^ ^]^ The energy calibration was conducted using an Ar‐ion sputter‐cleaned Au foil and calibrated according the Au4f_7/2_ transition located at 83.96 eV.^[^ ^144^ ^]^ Excitation energies were set individually for each core level to make sure a similar kinetic energy of the emitted electrons of 150 eV during measurements. Backgrounds of the acquired spectra were subtracted by using the Shirley method in CasaXPS,^[^ ^145^ ^]^ version 2.3.25. TGA and differential scanning calorimetry (DSC) were performed on a Mettler Toledo TGA/DSC 3+ from 25 to 500 °C at a heating rate of 10 °C min^−1^ under a nitrogen flow or an air flow of 50 mL min^−1^. The electrical conductivities of cobalt‐based thin films were measured using an in‐line 4‐point‐probe setup, recorded by a multimeter (U3606A, Agilent Technologies). Before the measurements, the probing spots on the samples were sputter‐coated by gold using a sputter coater (Q300TD, Quorum Technologies) with 30 mA current for 60 s, and the other parts of the samples were covered with a mask to avoid coating. SEM, AFM, TEM, and XRD investigations have been conducted on/from cobalt hydroxide and oxide samples deposited on silicon (100) wafers to avoid signal contributions from the substrate, while for profilometry, Kr physisorption, Raman spectroscopy, IRRAS, XPS, and NEXAFS, FTO substrates were used.

##### Electrochemical Measurements

All electrochemical experiments were conducted in a three‐electrode setup assembled in a Zahner cell (PECC‐2, Zahner) with a mercury/mercury oxide (Hg/HgO) electrode as reference electrode (RE) and a platinum wire ring as counter electrode. The reference potential of the Hg/HgO electrode was measured against a standard hydrogen electrode (SHE) (HydroFlex, Gaskatel) in the above‐mentioned electrolyte before the experiments and used as conversion value taking the Nernst equation into account. The working electrode (WE) was composed of cobalt hydroxide or oxide thin films deposited on FTO substrates. The geometric surface area exposed to the electrolyte was 0.785 cm^2^. 1 M KOH (pH ≈ 13.6) was utilized as electrolyte. The applied potential was controlled by a GAMRY Interface 1000 E potentiostat. LSV was acquired between 0.9 and 1.7 V versus RHE, using a step size of 1 mV and a scan rate of 10 mV s^−1^. The potentials in the LSV curves were corrected by 90% iR‐compensation through evaluation of the uncompensated (solution) resistance (*R*_s_) derived from fitting the EIS data using the EC‐Lab software (version.11.36). EIS was measured at 1.6 V versus RHE and the open‐circuit potential for applied frequencies between 100 kHz and 0.1 Hz using an AC modulation of 10 mV. Cyclic voltammetry (CV) was conducted between 0.6 and 1.5 V versus RHE with a scan rate of 100 mV s^−1^ and a step size of 1 mV for 50 cycles. To calculate the ECSA, the double layer capacitance was determined by EIS at the above‐mentioned potential. The double layer capacitances were derived from fitting of the Nyquist plots. CP was carried out by recording the changes in potentials between WE and RE at a constant current density of 10 mA cm^−2^ for 4 h. The electrochemistry experiments were carried out in the sequence of CV, EIS, CP, and LSV.

##### Statistical Analysis

The pore sizes of the samples were determined by evaluation of the top‐view SEM images using the Scandium software, and the sample size was 20 for each sample. Pore size distributions are presented in the form of ranges. The data of Raman, XPS, and NEXAFS (intensity) were normalized to [0,1] using Origin 2023 Pro before plotting. All collected data in the underlying study were evaluated and plotted with the Origin 2023 Pro software.

## Supporting Information

Supporting Information is available from the Wiley Online Library or from the author.

## Conflict of Interest

The authors declare no conflict of interest.

## Supporting information

Supplementary Material

## Data Availability

The data that support the findings of this study are available from the corresponding author upon reasonable request.
