# Supplementary material for: Nanocrystalline Ordered Mesoporous Co(OH)2 and Co3O4 Thin Films: Oxygen Evolution Reaction Activity from a Structural Properties Perspective
Source: Small Sci. 2025 Dec 13;6(1):e202500422. doi: 10.1002/smsc.202500422 (PMC12794680; doi:10.1002/smsc.202500422)
Supplement: Supplementary file 1 — Supplementary Material [file SMSC-6-e202500422-s001.pdf]

Supporting Information to

Nanocrystalline Ordered Mesoporous $Co(OH)_2$ and Co_3O_4 Thin Films: Oxygen Evolution Reaction Activity from a Structural Properties Perspective

*Qingyang Wu†, Stefan Lauterbach‡, Christian Dietz§, Achim Alkemper†, Lysander Q. Wagner┤├, Helmut Schlaad┴, Jan P. Hofmann† and Marcus Einert†**

†Surface Science Laboratory, Department of Materials- and Geosciences, Technical University of Darmstadt, Peter-Grünberg-Strasse 4, 64287 Darmstadt, Germany

E-mail: meinert@surface.tu-darmstadt.de

‡Institute for Applied Geosciences, Geomaterial Science, Technical University of Darmstadt, Schnittspahnstrasse 9, 64287 Darmstadt, Germany

§Institute of Materials Science, Physics of Surfaces, Technical University of Darmstadt, Peter-Grünberg-Strasse 2, 64287 Darmstadt, Germany

┤Institute for Physical Chemistry, Justus-Liebig University Giessen, Heinrich-Buff-Ring 17, 35392 Giessen, Germany

├ Center for Materials Research, Justus-Liebig University Giessen, Heinrich-Buff-Ring 17, 35392 Giessen, Germany

┴ Institute of Chemistry, University of Potsdam, Karl-Liebknecht-Strasse 24-25, 14476 Potsdam, Germany

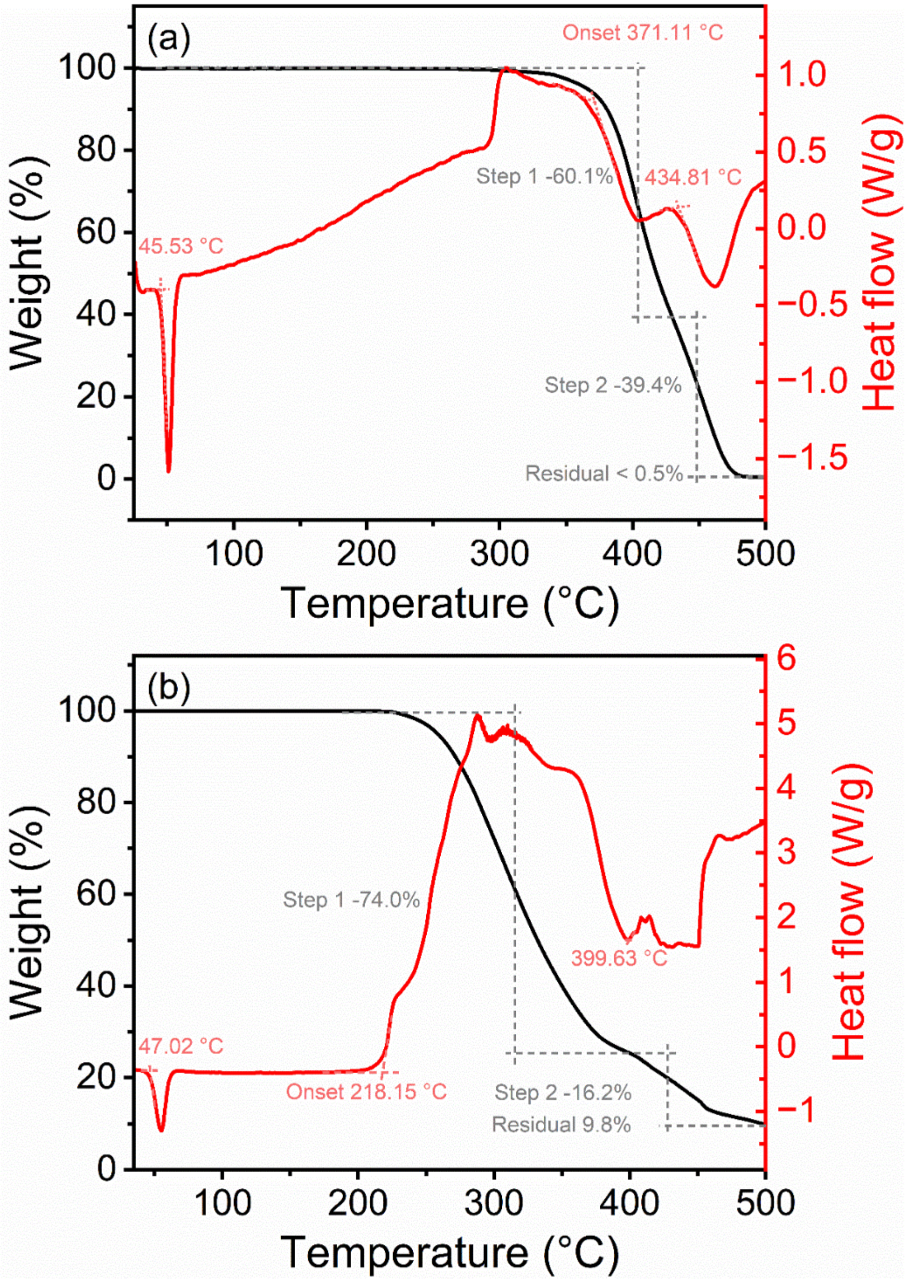

Figure S1. TGA (black) and DSC (red) curves of the KLE polymer heated from room temperature to 500 °C in (a) N_2 and (b) air.

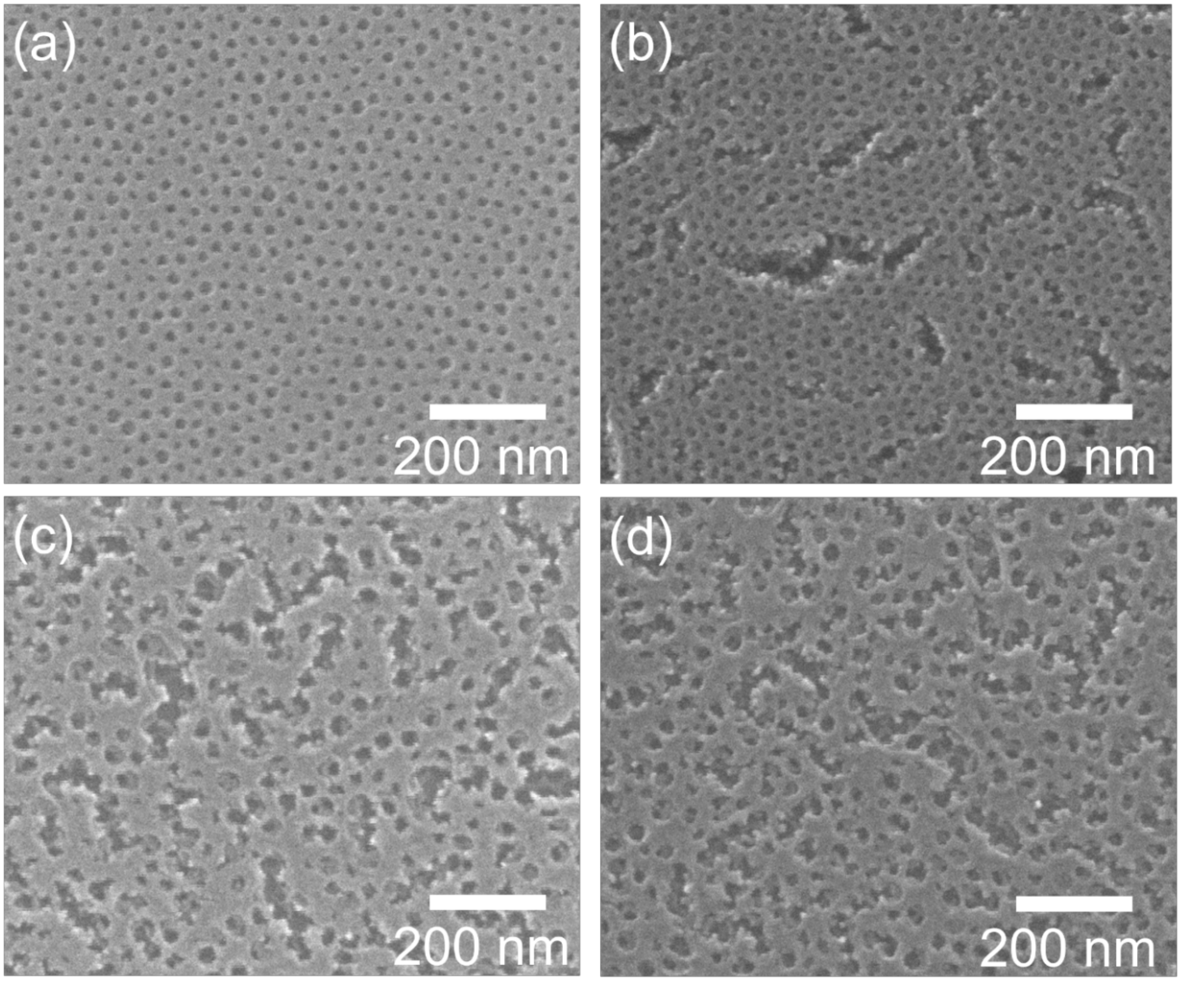

Figure S2. SEM images of cobalt-based thin films calcined at 300 °C for (a) 5 min, and (b) 30 min at 25% relative humidity. SEM images for samples prepared at (c) 9.5% and (d) 15% relative humidity and calcined at 300 °C for 30 min.

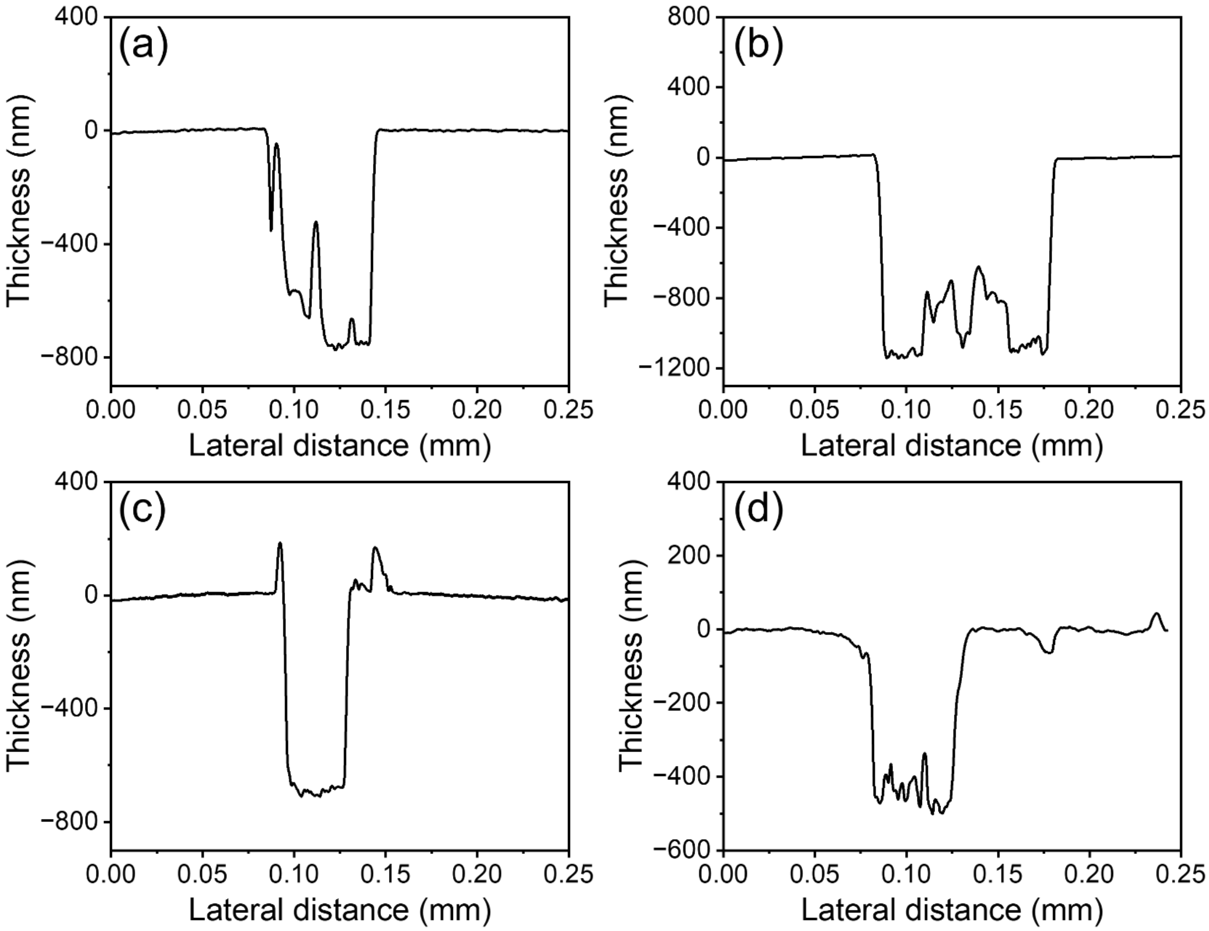

Figure S3. Profilometry scans for determination of the thicknesses of $Co(OH)_2$ and Co_3O_4 thin films calcined at (a) 200 °C, (b) 250 °C, (c) 300 °C, and (d) 400 °C.

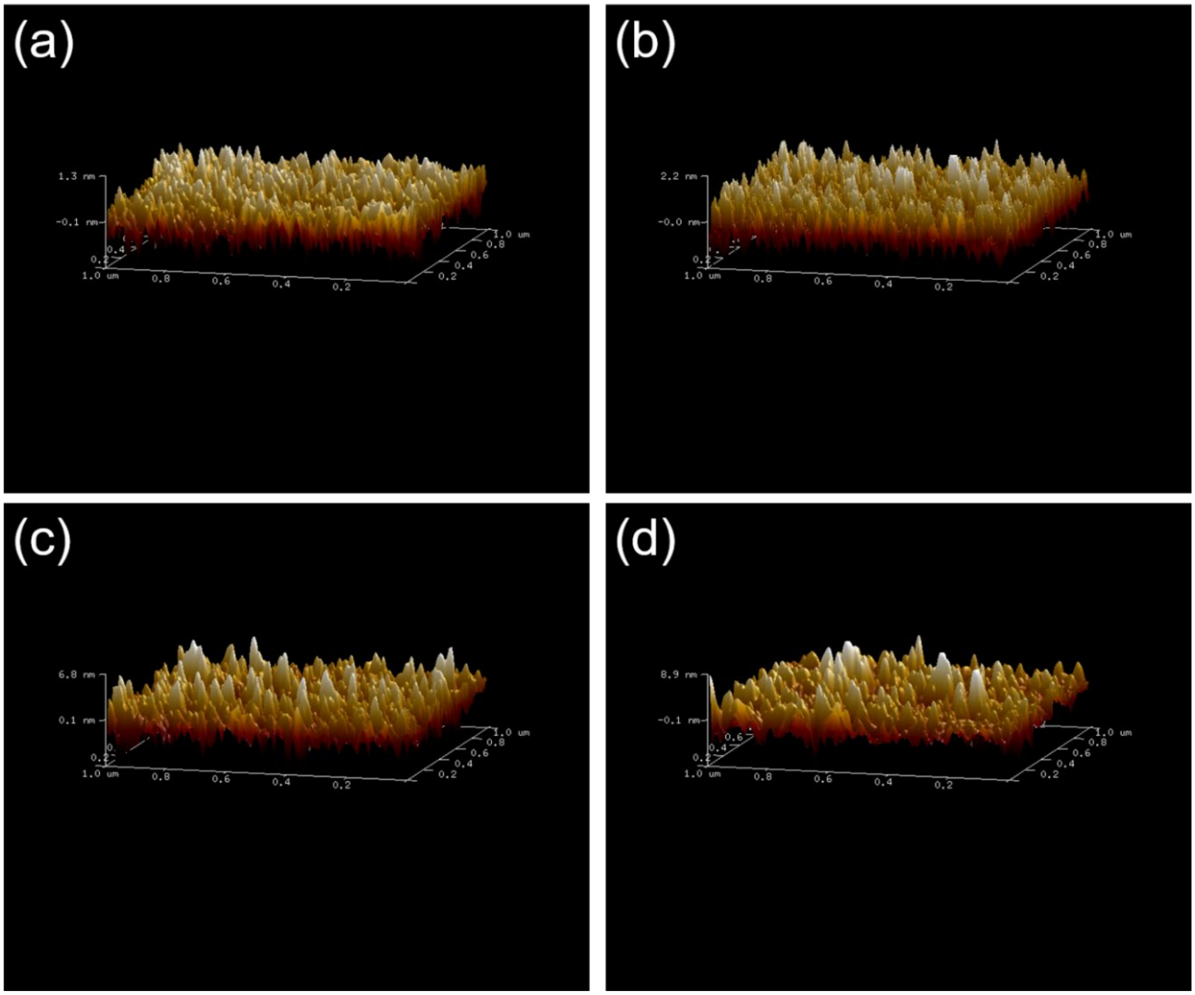

Figure S4. AFM 3D images of cobalt-based thin films prepared at (a) 200 °C, (b) 250 °C, (c) 300 °C, and (d) 400 °C for 10 min in air.

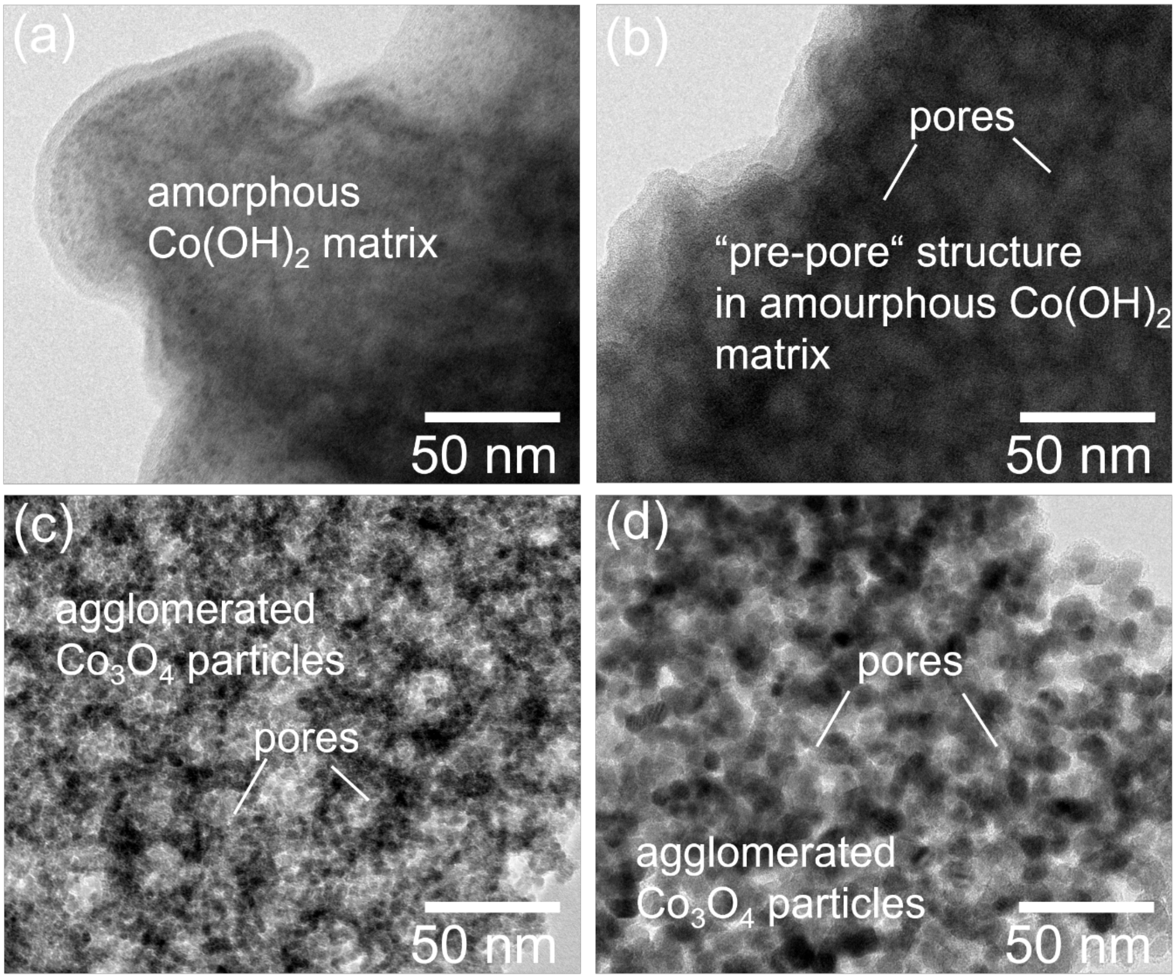

Figure S5. Bright-field TEM images of cobalt-based thin films calcined at (a) 200 °C, (b) 250 °C, (c) 300 °C, and (d) 400 °C for 10 min in air.

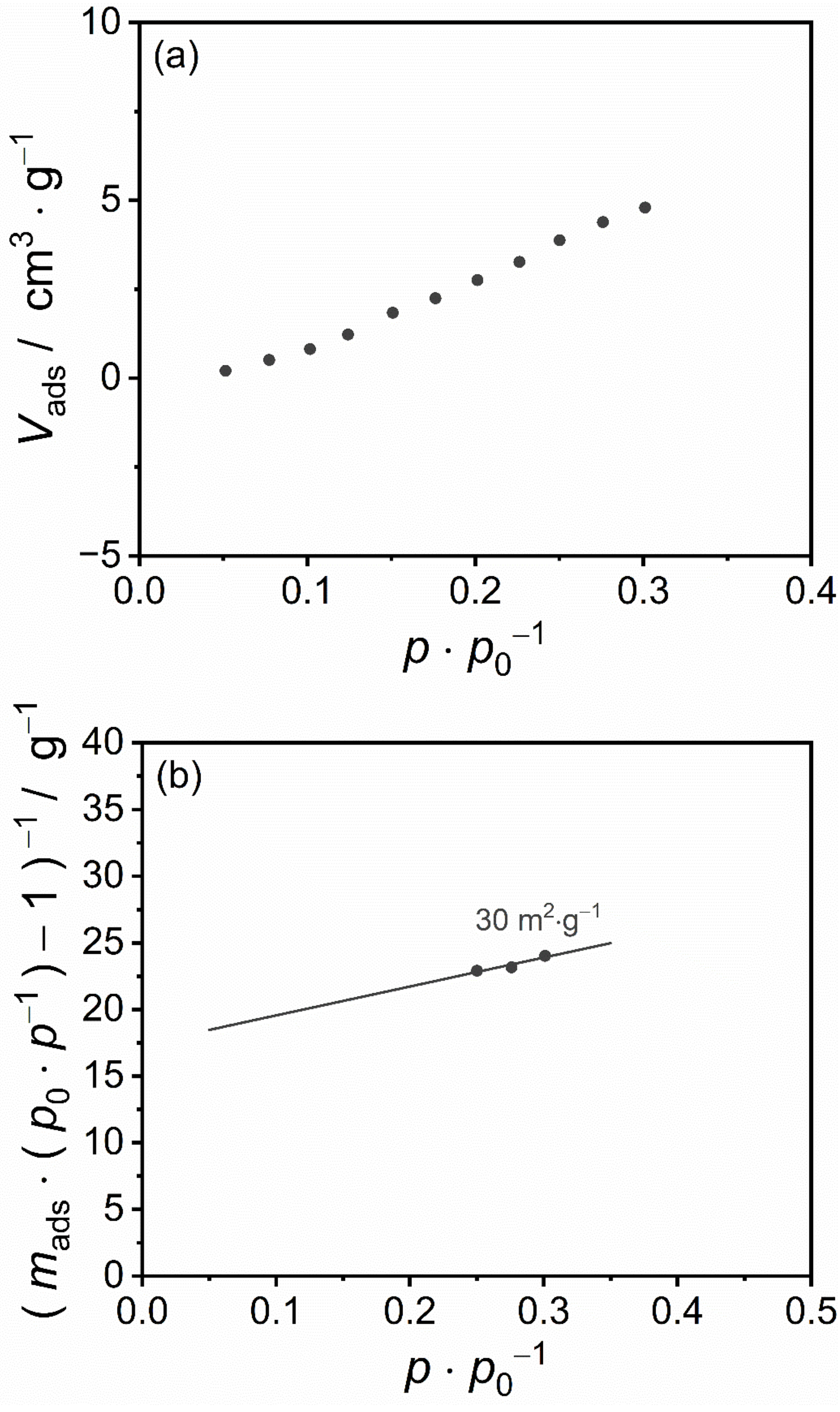

Figure S6. (a) Isotherms and (b) BET-plots of the mesoporous Co_3O_4-400 thin film investigated by Kr-physisorption.

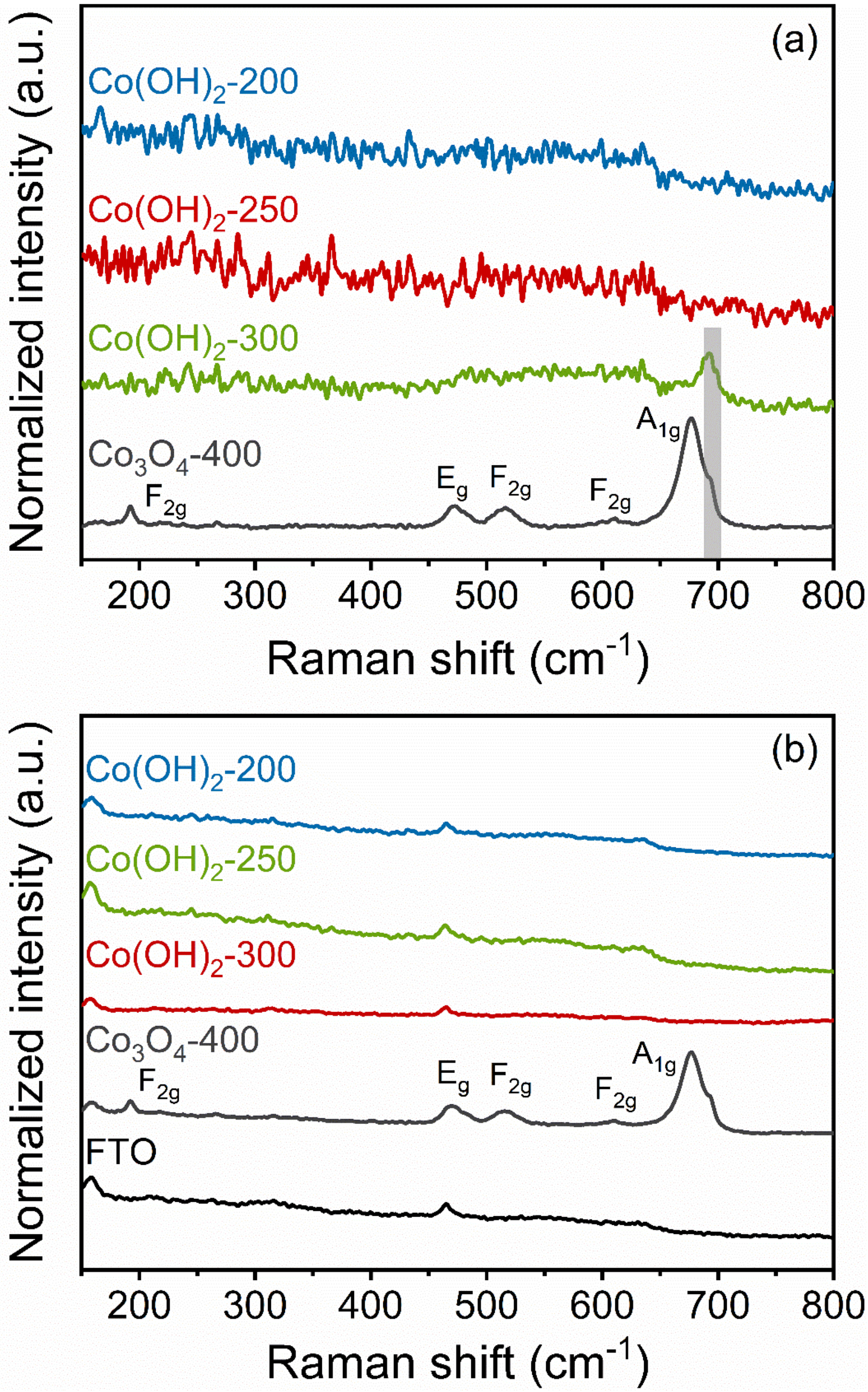

Figure S7. Raman spectra of cobalt-based thin films (a) with and (b) without substrate subtraction. The spectrum of FTO is taken as a reference.

Table S1. IRRAS vibrational bands of KLE and the corresponding assignments.

**Frequency (cm^{-1})**	**Assignment**	**Intensity**
2960	C–H asymmetric stretching	*s*
2856	C–H symmetric stretching	*s*
1465	C–H asymmetric bending	*vs*
1378	C–H symmetric bending	*m*
1346	C–H asymmetric wagging and CC stretching	*s*
1278	C–H twisting	*s*
1241	C–H asymmetric twisting	*w*
1143	C–O–C asymmetric stretching and C–C stretching	*s*
1060	C–O–C asymmetric stretching and C–C stretching	*m*
966	C–H rocking	*m*
843	C–H asymmetric rocking	*m*
721	C–H rocking	*w*
530	O–C–C asymmetric bending	*w*

vs, very strong; *s*, strong; *m*, medium; *w*, weak

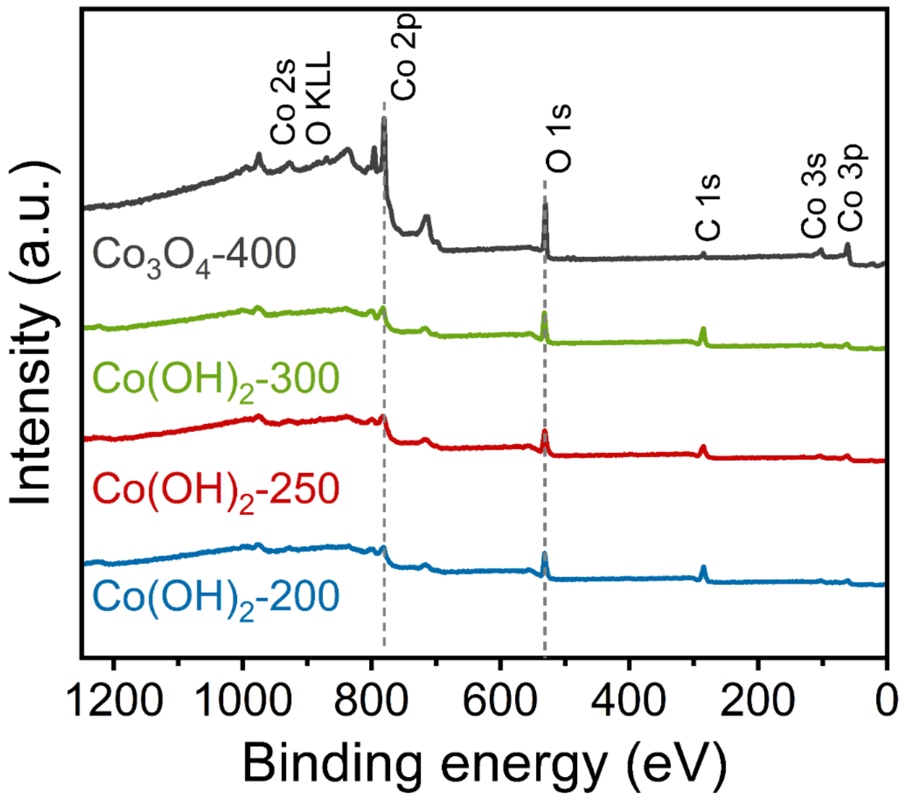

Figure S8. XPS survey spectra of cobalt-based thin films analyzed from 0 eV to 1250 eV, with the spectrum shifting according to the position of adventitious carbon at 284.8 eV.

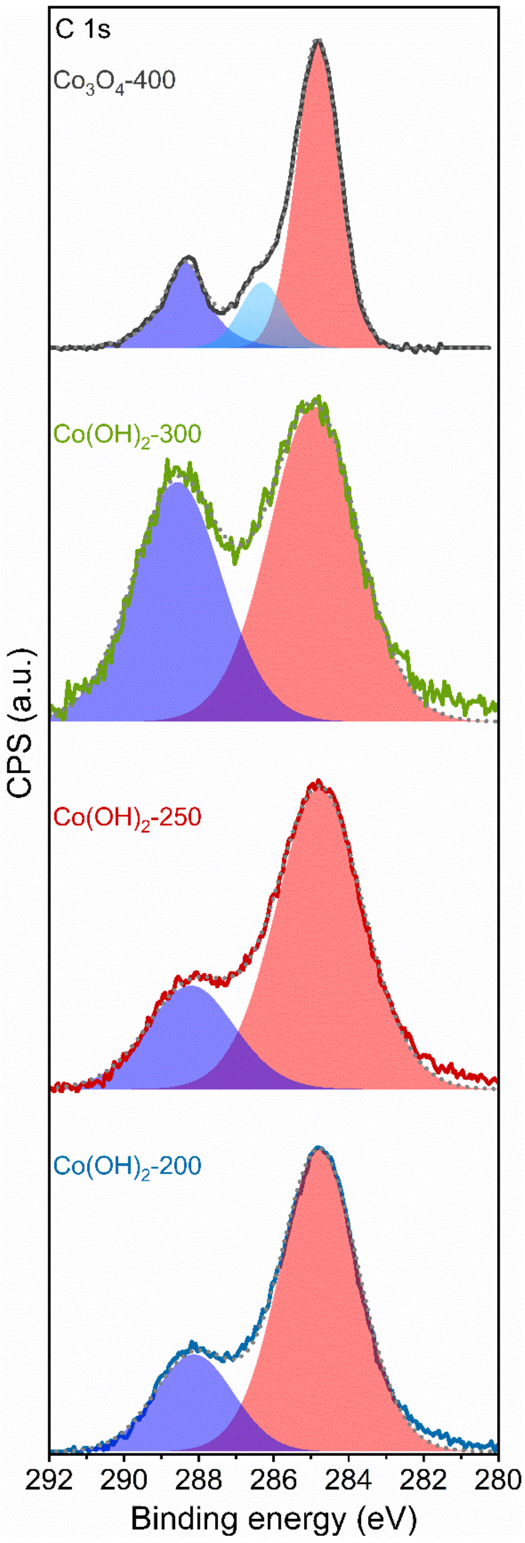

Figure S9. Detailed photoemission C 1s spectra of cobalt-based thin films analyzed by XPS and deconvoluted into adventitious carbon (*red* peak) and organic carbon (*violet* peak) species.

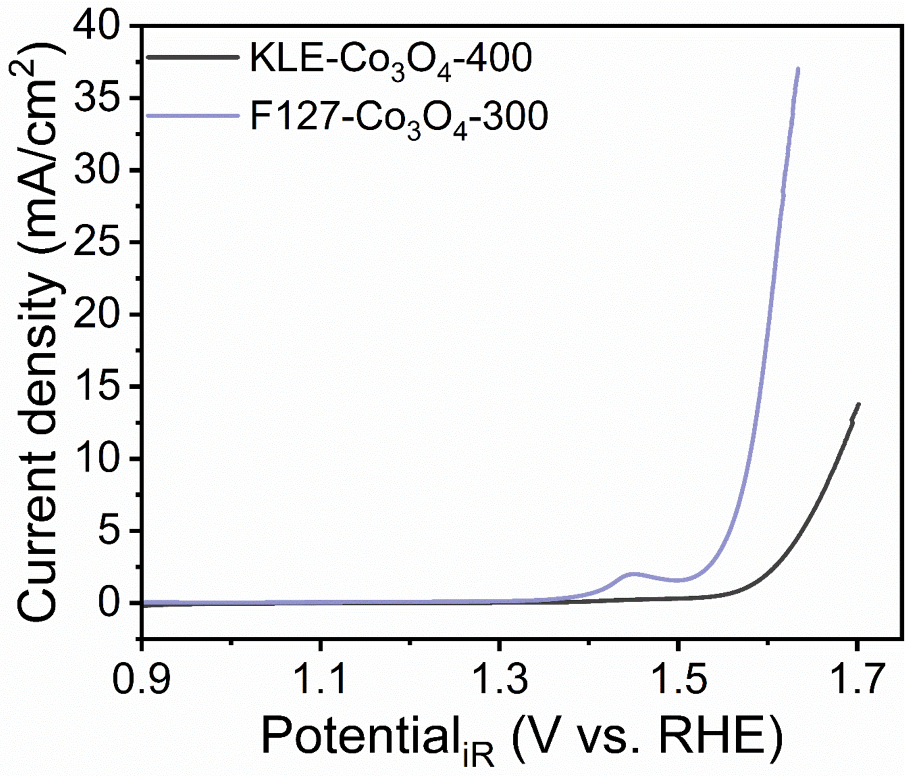

Figure S10. LSV curve of mesoporous Co_3O_4 thin films prepared via soft-templating with Pluronic® F-127 and calcination at 300 °C for 30 min from previous work.[1] The LSV curve of Co_3O_4-400 from the underlying work was added for comparison.

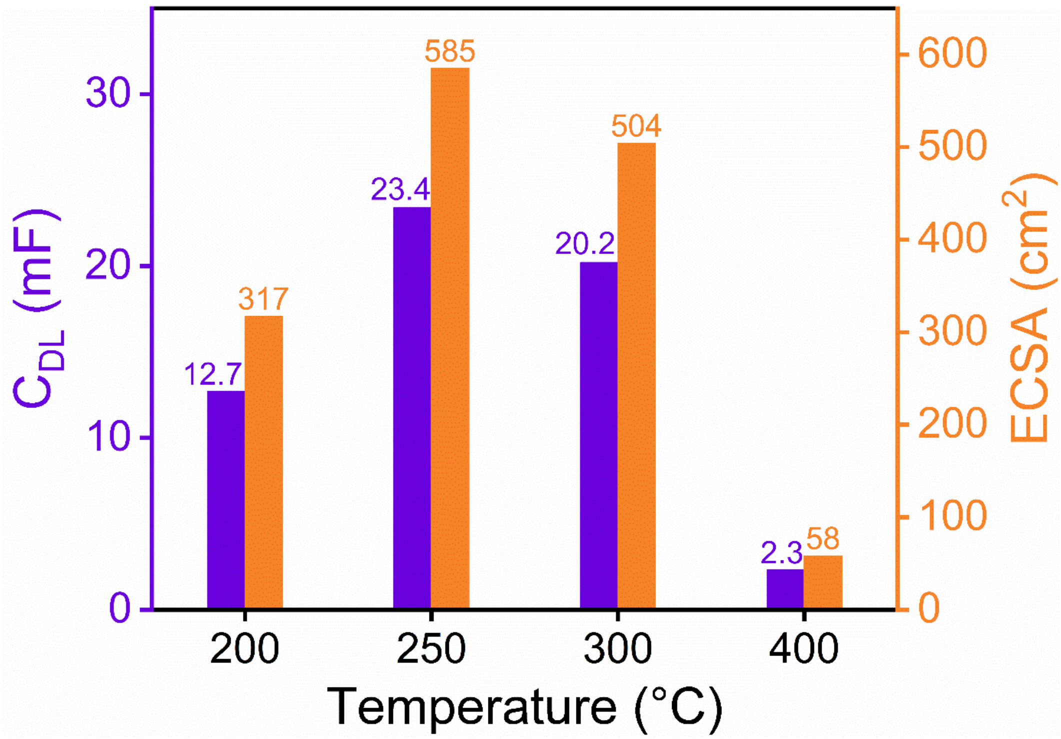

Figure S11. Column plot of C_{DL} and corresponding ECSA versus calcination temperature of cobalt-based thin films.

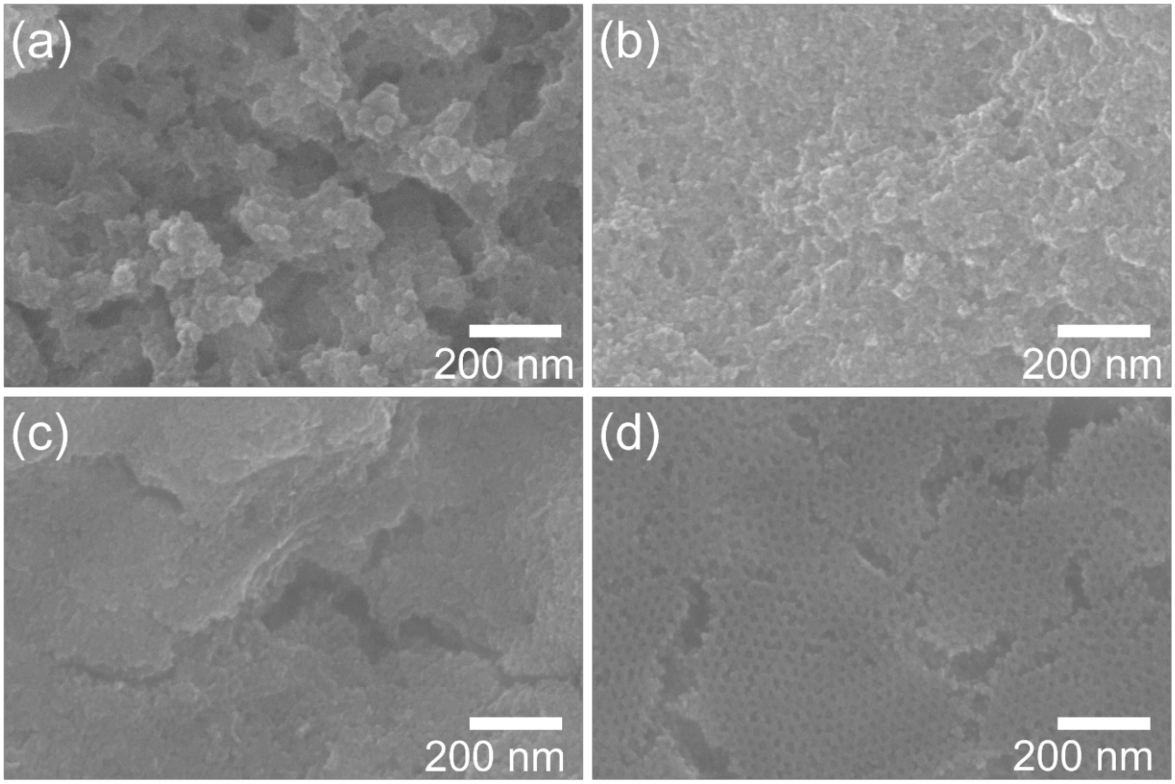

Figure S12. Top-view SEM images of cobalt-based thin films calcined at (a) 200 °C, (b) 250 °C, (c) 300 °C, and (d) 400 °C for 10 min and after performing electrochemical experiments (post-analysis).

Reference

1. Wu Q, Mellin M, Lauterbach S, et al. “Soft-templated, mesoporous Co_3O_4 thin films for electrocatalysis of the oxygen evolution reaction.” *Materials Advances* (2024):5(5):2098–109.
